# Clinical Review and Hospital Reports

**Published:** 1841-01-01

**Authors:** 


### Clinical Review.

### ROYAL WESTMINSTER OPHTHALMIC HOSPITAL.

Report on the Result of the Operations for the Cure of Squinting, performed at the Royal Westminster Ophthalmic Hospital, between the 18th April, and 30th October, 1840. By Charles W. G. Guthrie, Jun. Assistant-Surgeon to the Hospital, &c. &c.

In our last Number, we noticed the interesting Report on the Operation for Squinting, by Mr. Charles Guthrie, jun. The present is a pendant to that; and, independently of containing the results of former operations, it adds some new facts, both curious and important, to the stock. We shall notice the principal points.

1. *Safety and Success of the Operation.*

"Near seven months have elapsed since it was first introduced into the Hospital and into this country, since which time I have done 340 operations for internal, or converging squinting, and 16 for external or diverging squinting. In no case has any inflammation of the ball of the eye followed the operation, neither has inflammation of any other part ensued, requiring further treatment than the application of a bandage and cold water for the first twenty-four hours. In by far the greater number of cases the eye has been exposed the morning following the operation, a dose of aperient medicine has often been omitted, poor patients have frequently gone to their work the next day, and in no one case has the slightest bad consequence followed this operation. The success has been, in cases of internal squinting, complete, and although some few have given much trouble, I have I believe arrived at that point which permits my venturing to say, that I have met with no case which is not curable; and that although in two or perhaps three cases the cure has not been as perfect as I could desire, it has been because the patients have hesitated in complying with my wishes."

2. *What is to be done if the Eye resumes its Cast.*

"When in internal squinting the operation does not perfectly succeed, the eye is sometimes perceived to resume a little of its original cast, or squint, from the eighth to the fourteenth day, or about the time, I conceive, at which the muscle becomes re-united to the ball of the eye, or to some part within the orbit, and considerable anxiety is felt by both surgeon and patient at its occurrence. It does not however follow, that the operation will not eventually succeed, and the eye be restored to its proper place by the efforts of nature alone. I have seen it do so several times, nevertheless I do not now trust to the unassisted efforts of nature when I perceive the slightest turn beginning, but direct the other eye to be padded, or kept closed, so that its vision may be from time to time effectually prevented, and the patient to use, or to try to use the remaining muscles. I do this, I believe with success, even when the eye has a turn in the opposite direction after the operation, relying on the efforts Nature will make to bring the axis of the single eye actually in use, (whether the other is covered or not,) into its proper place, even although one of the muscles, usually supposed to effect this object should have been divided; and this education of the eye is of great service, even where the padding or closing of the other is omitted.

In some obstinate cases of long standing, the eye, almost immediately after the division of the muscle, returns to nearly its pristine state of obliquity, and although the division and separation of the muscle and its attachment in every direction, in the manner I have especially indicated, until the outer or sclerotic coat is fully exposed to a considerable extent, enables us to succeed, in most instances, in effecting the object in view, it does not always do so; and in a few cases I have been obliged to desist without bringing the eye to an exact central position; or I have found it a little turned inwards on the next or some other succeeding morning. The remedy for this evil, and which generally succeeds, is to exercise the eye in a regular manner, of which Charles Marten, 18, Lisle Street, is an excellent example, and in such cases the eyes will usually be found to be naturally convergent. It may occur when the incision in the conjunctival membrane unites in the first instance by adhesion, which it will do in some cases when it is small, and the eye by turning at all towards its former oblique state, facilitates the process. I suspect also that the ball of the eye rests in its peculiar bed of fat and membrane, like a cast in a mould; and that when the eye is liberated from its squinting state, it does not remain in its new and partially unsupported position, but gradually falls back into the mould of fat and membrane originally formed around it. I am led to apprehend that the conjunctival membrane materially assists from its general attachment to the eyelids, in maintaining the eye in its old position, from which it is, however, gradually drawn in these cases by the efforts of nature assisted by art."

3. *When the Operation is followed by Squinting of the other Eye.*

"When one eye has been operated on, and restored to its proper state, and the patient is sensible he can turn it in every direction, and has so far recovered his sight as to make use of it, the other with which he knew he had an occasional cast, sometimes begins to squint more frequently, and this may become a permanent defect. In such cases, and I have alluded to them in the last edition, p. 4, there can be no hesitation about the propriety of operating on the second eye, and which succeeds in restoring both to their proper situation.

Whenever I can perceive this state to exist to an extent which I am satisfied from experience, is not likely to be removed by any management of the eyes after one operation, I recommend the patient to have them both done at once, or with only the interval of a day, so that the education required for both in seeing in a new way may go on simultaneously; and which I have found to be of great advantage in many instances."

4. *When the Patient Squints with both Eyes.*

"In some rarer cases it will be found that the patient squints with both eyes, although he is only aware that he does so with one; and neither he nor his friends will believe it; and I now apprehend that these are the cases in which, when the operation does not fully succeed at once, it has been often thought necessary to divide, or attempt to divide, other muscles than the one originally intended to be operated on; whilst the incisions into the orbit and through the conjunctival membrane are unnecessarily prolonged, giving rise perhaps to those great protrusions of the eyeball, or to the loss even of eyes, both of which evils, it has been stated, have followed these operations, but which ought never to occur, and which I hope these observations will in future prevent. It is a difficult thing to persuade, much more to convince a person that he squints with both eyes, when he cannot see it himself, nor any of his friends can see him do it, although the defect in one eye is apparent to every body. Under these circumstances he submits to an operation on the eye he knows to be affected, which is easily done: but when the surgeon examines it carefully after the operation is over, and the patient tries to use it, he finds that he can still turn it inwards, and the operator sometimes thinks it necessary to cut in various directions until the eye is sufficiently loosened in the orbit to lie in the desired position. A more cautious operator is contented to have removed the greater part of the evil, trusting to the management of the eye afterwards for its complete recovery. This was my practice, and although I succeeded in many instances, still I have found among the cases of 30, 40, and 50 years' standing, some that I could not entirely overcome. These persons have presented themselves, for the most part during the last two months, the more elderly people having begun to think that they might as well avail themselves of the advantages the operation afforded; and in the instances alluded to the patients all persisted that they squinted only with one eye, and which they readily submitted to operation; but when told they must have the other done, they as manfully resisted.

In my last, or fourth edition, I alluded to this, as I have before remarked, but I did not like to express myself more fully on the practical point, until, from repeated observations, I was quite satisfied of the fact I intended to inculcate. In such a case, that is when the eye still turns in after the operation, and I am satisfied I have done all that ought to be done, I do not persist in trying to do more, but at once operate on the other eye, whether it apparently stands in need of it or not; and I ascertain the fact of the necessity in the following manner, if it had not been done before. The bleeding if any having ceased, I desire the patient to look me steadily in the face with the eye which has just been operated on, whilst I keep the other closed. When this has been done for a few seconds, I gradually raise the lid of the supposed sound eye, and in every instance of the kind, have found it turned inwards, so much so as to cause the greatest surprise to the friends of the patient, or other bystanders, and, through their observations, as well as my own, the person at last submits to have the operation done on that eye also. It is much better they should be done at the same time, when both eyes immediately become straight, but this does not always follow so instantaneously if there is a long interval between the operations, and the first eye may require some education afterwards. But I believe that, with this improvement, the cure of an internal squint may always be accomplished."

5. *When a Patient fancies that he Squints.*

"The imagination has exercised its usual activity even in this complaint, and persons have been found who were so perfectly satisfied they squinted badly, that they earnestly desired to have an operation performed, although the obliquity was scarcely discernible. In such cases, and indeed in those in which the second eye is operated upon, when the persons were unconscious they had any deformity, I merely divide the tendon without any of its lateral attachment; thus weakening rather than destroying its power."

6. *External Squinting.*

"The operation for the division of the *external* rectus or straight muscle is as easily done as that for the internal one; but the result in external squinting is not always so satisfactory. It has been said that this operation does not succeed; that, strictly speaking, none have been successful, although all the cases operated upon have been improved in a trifling degree; in fact, that they only squinted a little less than before. This, and all the other observations I am acquainted with, are on many points erroneous, although in some few they may be correct.

I have however met with four cases in which this happy result did not follow to the same extent. In the first, I operated on the right eye, and brought it to the centre, but it never would turn in, the internal straight muscle seemed to have no power over it, and as the man thought himself very much improved, and did not wish anything more to be done, he absented himself from the hospital, and I have not been able to find him. The second was a perfectly similar case—the third I have under treatment, and think I shall cure—the fourth had both eyes operated upon in the country for internal squinting by the division of the internal straight muscles, and the consequence was that both eyes turned outwards. The surgeon then divided the two external straight muscles, but the eyes again turned outwards, causing two very disagreeable casts at the same time. I have redivided these external muscles further back than before, and the right eye is cured, the left is nearly so, although a trifling cast, compared with what it was before, remains, but the ultimate result of further time and treatment are however to be ascertained. The next case I shall adduce is perhaps the most extraordinary, and will give the most satisfaction of any I suspect that have yet been reported.

Thomas Williams, aged 17, of No. 15, Crown Street, Westminster, applied on the 24th August with an internal squint of the right eye, which was operated on the same day in the usual manner, without any thing remarkable being perceived. The next day it appeared rather more out than in, and this gradually increased, so that, although he could frequently look very naturally with it, and particularly when he made an effort to do so, it was evident that it turned a very little outwards when perfectly quiescent. On trying the other eye, which he declared to be perfectly sound, in the manner I have directed to be done to ascertain its state, when the eye which has been operated upon is intently fixed on the face of the experimentalist, I found to my surprise that it was on every trial directed outwards. I informed him that the only way to make the right eye quite straight, and to turn in, was by dividing the *ex*ternal straight muscle of the left or sound eye. To this he demurred for some time, but at last consented, on the 28th of October, and I cannot express the pleasure I felt on finding, after the operation, that the train of reasoning which induced me to do this operation was crowned by success."

We are all very much indebted to Mr. Charles Guthrie.

### GUY'S HOSPITAL.

GUY'S HOSPITAL REPORTS, No. XI. Edited by GEORGE H. BARLOW, M.A. and L.M. Trinity College, Cambridge; and JAMES P. BABINGTON, M.A. Trinity College, Cambridge, Member of the Royal College of Surgeons.

THE present Number of these valuable Reports presents us with the following Papers:—

Remarks on the Report of Syphilitic Cases (continued); by C. Aston Key. On the Treatment of Incipient Phthisis; by H. Marshall Hughes. On Disorders which are Variable, and on the Practical Inferences which are deducible from the Character of Changeableness; by T. Wilkinson King. On the Episternal Bones occasionally found in Man; by T. Wilkinson King. On some Supplementary Muscles of the Anus, described by Dr. Horner of Philadelphia; by T. Wilkinson King. Case of Transposition of the Aorta, Trachea, and Œsophagus, Tuberculated Liver, and Scirrho-Cancer of the Rectum; by Henry Ewen.—Communicated by C. Aston Key. On the Forms of the Cartilages which keep open the principal Divisions of the Bronchial Tubes; by Jonas King. Case of Urinary Calculi, formed on a piece of Straw; by Henry Norris. —Communicated by Sir Astley Cooper, Bart. On the History of a Supposed Hermaphrodite; by Robert Merry.—Its Dissection by Sir Astley Cooper, Bart. Practical Hints on the Treatment of Stricture of the Urethra; by Bransby B. Cooper, F.R.S. Operation for the Radical Cure of a Reducible Inguinal Hernia; by Bransby B. Cooper, F.R.S. Case of Cerebral Disturbance, dependent upon Disease of the Pericardium; by Dr. Yonge.—Communicated by Dr. Bright. Observations on Diabetes: with Cases illustrative of the Efficacy of Ammonia in the Treatment of that Disease; by George Barlow, M.A. & L.M. Observations on Abdominal Tumors and Intumescence; illustrated by Cases of Diseased Liver; by R. Bright, M.D. F.R.S.

As we believe our readers prefer the practical to the rare, the useful to the curious, we shall pick out the papers of the former class for notice first, and allude to the remainder afterwards. We shall commence with our able and excellent friend, Mr. Bransby Cooper.

### Practical Hints on the Treatment of Stricture of the Urethra; By Bransby Cooper, F.R.S.

Mr. Cooper has already communicated one Paper on this subject to the Profession, a paper which we noticed at the time. The present is a continuation of *that.* An object of both has been to deprecate the employment of force in the introduction of instruments. In the paper before us, Mr. Cooper enters more into detail on the means of treatment than he had prevously done.

" On first examining," says Mr. C. " a stricture, either a silver catheter or a bougie may be used for the purpose of ascertaining its seat and condition. No. 6 is the best-sized instrument for this object: it is nearly of the size of the urethra, and is much less likely than a smaller one to get entangled in the lacunæ of the canal. When the instrument is first passed down to the stricture, gentle pressure should be exercised, and, if its passage be resisted, should be continued for a few minutes. If the obstrction be owing to muscular spasm, continued pressure of three or four minutes will be sufficient to overcome the contraction: if after that time, however, the obstacle does not yield, its persistence evidently depends upon the density of an adventitious deposition. Where, in such a case, no great pain is experienced by the degree of pressure hitherto employed, and no bleeding occurs, force may be used, to the full extent considered safe by the surgeon: but if this is still ineffectual, no further mechanical means should be had recourse to (unless the bladder be much distended, and the symptoms of retention urgent), but leeches to the perinæum and a purgative draught should be prescribed. It generally happens, in cases of no very long standing: that great benefit is at once derived from this treatment; and I have frequently seen the patient recover after it has been adopted for three or four alternate days; but in cases of older date, great difficulty of micturition is often found to remain, in spite of it. To combat this symptom, the application of the bougie should be continued; as it is now ascertained that the difficulty of directing the instrument into the bladder depends upon the density of the stricture, and not upon muscular contraction. A new indication now therefore presents itself, viz. the removal of the adventitious matter: this is most readily effected, by maintaining an equable degree of force upon the surface of the stricture, which induces inflammation and softening-down of the newly-formed substance. In the course of a few days, suppuration will be found to have been set up; after which, five or six repetitions of the application of the instrument will be sufficient to effect its passage through the obstruction; and thus will be performed a cure of stricture in what I may term its second stage.

It is in this second stage that the precaution which I have alluded to above is necessary; for it is requisite to be as cautious with respect to the precise direction given to the instrument when thus gently applied, as when force is used to push the catheter or sound into the bladder; inasmuch as, without proper attention to the direction of the instrument, a false passage is as likely to be produced in the one case as in the other. This I shall shortly be able to shew, by relating cases in point. If gentle pressure be repeatedly made against the side of the urethra, instead of upon the stricture itself, the mucous membrane naturally yields to the continued application of the instrument; and a new passage is gradually produced, which by violence is frequently effected at once. When an opening has thus been formed in the urethra, it is not a necessary consequence that any train of symptoms should at once point out the error which has been committed. On the contrary, from the comparative facility with which the instrument passes onwards towards the bladder, the surgeon fancies, at first, that he has overcome the true source of obstruction, and indeed frequently succeeds in reaching the bladder with his instrument, having pushed it again into the urethra, or through the prostate gland: a flow of urine then ensues, which gives him reason to suppose that the cure is complete. On the other hand, however, the urine is sometimes extravasated, as soon as the false passage has been made; an abscess is formed in the perinæum; and all the symptoms follow inseparable from such a state of things, requiring at once the laying open of the perinæum, and the evacuation of the extravasated urine; and leading to the abandonment of the gentle mode of treatment, for the cutting through of the permanent stricture,—an operation which I have before described."

Perhaps it is going rather far to say, that no force and force are equally likely to occasion a false passage. On the *gutta cavat lapidem* principle, the frequent mal-application of a catheter, with gentleness, may send it out of its track, yet still we should doubt its doing so as frequently as the violent employment of the instrument.

Mr. Cooper's opinions are necessarily possessed of weight, and ought to influence the surgical world. Yet as sentiments and practice differ, we are ourselves disposed to give the preference to another method. If a patient presents himself with stricture, we ascertain, as well as may be, the dimensions of his stream of urine, if he makes one. And we take an instrument, a gum or a metallic catheter or bougie, about that size, or rather smaller, and attempt its introduction, generally with success. If the water only dribbles and the stricture is a bad one, we must say that we are advocates for beginning with the cat-gut or the wax bougies of the smallest size. In many instances, to say the least, this may be introduced, and if it is, the rest is but a matter of time. If it cannot be got in, the gum or silver catheter is in reserve. Should that be small or large? Our predilections are for the small. The metallic one should be employed with delicacy and with address, no doubt, and force is seldom justifiable. But, so far as we have seen or can determine, the small instrument, gently used, is safe and serviceable, more so, indeed, than the large.

Mr. Cooper admits, what we fear is too true, the frequency of false passages. If our own experience is to be trusted, violence has, in almost all instances, been father to them. Mr. Cooper remarks:—

"In cases where a lengthened false passage has been made into the bladder, and where no extravasation of urine has followed, it becomes very difficult for the surgeon to ascertain, with certainty, that the fistulous passage exists: and I believe that this can only be done by those who are in the habit of frequently passing instruments into the bladder. As, however, such passages are frequently made in the upper region of the urethra, their false course may be ascertained from the fact, that a straighter intrument than the one usually employed will pass through them with facility; and that when in the bladder, the instrument wants the freedom of motion exhibited by the catheter, which has been passed through the urethra only. If the false passage has been made in the lower part of the urethra, the instrument will be found to move in the segment of a larger circle than usual; and its escape from the urethra can always be ascertained by passing the finger into the rectum. False passages of the lower region are generally the result of the forcible use of the instrument for strictures in or near the bulb; while those of the upper part are a consequence of the continued pressure made upon the obstruction, with the penis drawn forward to fix the point of opposition. In both of these cases, although the patient, from the absence at first of any difficulty in emptying his bladder, may imagine himself permanently relieved, he is doomed to be very quickly disappointed: should the surgeon allow only a few days to elapse without passing the instrument, the obstruction to its progress, and the difficulty in voiding the urine, recurs; for the new canal is incapable of remaining many days pervious, without the frequent passage of the catheter; unless, indeed, a mucous lining membrane is formed within it; to produce which, a considerable length of time is necessary. A case of this kind occurred to me; in which, after having passed the instrument for several weeks, in the belief that I had re-established the natural canal, it at length, on a sudden, took a new course into the bladder; and this proved to be through the natural urethra. Probably this took place from the stricture having been spontaneously cured by the removal of the irritation from the urine, which for some time previously had passed through the new canal. The patient subsequently suffered no inconvenience from the false passage which had been made; nor had I ever, after the discovery, any difficulty in passing the instrument into the bladder through the natural canal. I cite this case, not because I believe it to be of rare occurrence, but merely because the discovery of it is uncommon. I think, indeed, these false passages to be so frequent, as to be present in most of those cases where difficulty of passage continues whilst the patient is under treatment: at all events, post-mortem examinations of the urethra, in cases of stricture, shew that these deviations from its natural course are extremely common."

When we have had reason to suspect the existence of false passage, (and a good anatomist can scarcely fail to *suspect* it sooner or later, if there is one) we have seized the opportunity (if it offered) of an instrument reaching the bladder by the true one, and confined the patient to bed, with the instrument tied in. So soon as it is loose and suppuration is freely established, a larger one may be substituted for it, and by this means the proper canal of the urethra may be dilated with rapidity. Elastic catheters answer best. It is a common practice and we merely mention it in connexion with this particular case.

*Caustic.*—"In cases of stricture which resist the application of the common bougie or catheter,—for sometimes, owing to the great density of the adventitious substunce, these instruments will not succeed in causing the latter to soften down, even though they may be used for several days or even weeks in succession,—in such cases, the caustic bougie must be had recourse to; not merely as an escharotic, to decompose the part, but that it may, by its peculiar action, induce inflammation, softening down, and suppuration. A successful result generally follows a few applications of this instrument: and although in some cases it may be delayed, I have never seen it fail to produce, sooner or later, a beneficial effect. There are occasions in which its efficacy is exhibited with singular rapidity. For instance, in a case of obstinate permanent stricture, for which I had been for several weeks attempting to pass instruments without success, I at last easily succeeded in introducing a catheter into the bladder, after passing eight times only the caustic bougie; and the patient ultimately recovered and has never had any recurrence of the disease.

The caustic bougie is also of the greatest benefit in irritable stricture, which is always attended with bleeding on the gentlest application of the instrument, and with frequent sudden spasms, occurring without any apparent cause, beyond slight exposure to cold and damp, or some excess in the use of wine or spirits, and which render micturition difficult. I need scarcely observe, that the cautions I have described above, as to the precise application of instruments to the stricture itself, are most especially to be observed in making use of the caustic bougie; or that a false passage is very likely to be produced, if it be made to press and act on the urethra itself, instead of on the adventitious matter."

Mr. Cooper relates three cases in illustration of the preceding observations, and of the benefits derived from the use of the caustic bougie. They do not appear to us, we must confess, conclusive, and, having at one time, seen a good deal of the application of caustic to strictures, our opinions of it are not quite so favourable as Mr. Cooper's. These are points, however, on which, perhaps, surgeons will never be perfectly unanimous.

Mr. Cooper relates several cases in which the operation of cutting through the stricture was performed. They ended very satisfactorily.

The profession ought to feel indebted to Mr. Cooper for these useful communications.

### ON THE TREATMENT OF INCIPIENT PHTHISIS. By H. MARSHALL HUGHES, M.D.

Dr. Hughes remarks that, from the pathological investigations of Laennec and others, it has, of late years, been considered possible that tubercular cavities, after collapse and contraction, may become effaced by the adhesion of their parietes; or that, from their limited extent and inactivity, they may cease to be a source of danger, or even of inconvenience; and therefore, that if tubercles exist not in other parts of the lungs, the cure of advanced phthisis may be spontaneously effected. The morbid anatomy of the parts upon which this opinion has been founded, may, in some instances, have been investigated with too little care, and the supposed facts may have been received upon insufficient evidence; but that tubercular cavities may contract, collapse, and become inactive and innocuous, is a belief too securely based, and too strongly supported, to be materially affected by the doubts expressed in the recent work of M. Fournet. The cure, however, of phthisis, in its advanced stage is, at all events, extremely rare, and the influence of art on it erratic and indeterminate.

But though, continues Dr. Hughes, when tubercles are numerous or softened, or when large excavations exist in the lungs, we yet need remedies to stop the progress of the disease, there appears to be no sufficient reason for believing that scrofulous matter may not be absorbed, or so altered as to cease to be a source of irritation in the lungs, as well as in other parts of the body, as the glands of the neck or mesentery; and therefore that phthisis, in its early stages may not be cured, or at least indefinitely suspended. That, by the early adoption of general as well as local treatment—of hygienic combined with medical means—the incipient disease may be subdued, and that the further deposition of tubercles may be prevented, is an opinion which is now general, and rapidly increasing.

Dr. Hughes, after adverting to Sir James Clarke's excellent treatise on consumption, and to Dr. Carswell's views of the nature of tubercle, thus professes his own creed :—

"Tubercular phthisis, then, I believe to be a constitutional affection, either hereditary or acquired, in which an unorganizable matter is eliminated from the blood in a fluid state, together with the natural secretions of the parts affected. I suppose that this matter becomes at length solid, by the absorption of its fluid constituents, and either remains upon, or is more or less quickly removed from the surfaces on which it is deposited, according to the entire want or to the freedom of communication of those surfaces with the natural outlets of the body;—that its principal seat is the free surface of the mucous and serous membranes;—and that its most frequent locality, when affecting the lungs, though occasionally infiltrated through all the tissues of the organ, is the mucous membrane of the air-cells and smaller bronchial tubes. I moreover believe that this deposit or secretion, though it not unfrequently occurs without any decided or observable increased action of the vessels of the lungs, and is therefore not at first characterized by any marked thoracic symptoms, is, when the predisposition already exists, often induced, promoted, and increased by bronchitis, and other inflammatory affections of the tissues, by the vessels of which it is secreted."

Dr. Hughes does not so much allude to the management of the constitutional affection, but rather confines himself to the treatment of the pulmonary disease. He first alludes to the principal remedies he has employed.

1. *Emetics.*—At first Dr. Hughes prescribed them only in advanced phthisis—latterly in the incipient. He has tried many and various forms. The following are the results of his experiments.

"I believe that either the simple sulphate of zinc, or ipecacuanha, in doses of twelve grains, or a combination of six grains of ipecacuanha and two of sulphate of copper, are the most desirable forms and proportions that I have used. Smaller quantities have occasionally failed. Under these circumstances, nausea, but no vomiting, and on one or two occasions diarrhœa, have been induced by the medicine. The emetics have been directed to be taken in a few ounces of warm water, about an hour before breakfast, every morning; or every second, third, or fourth day, according to the strength of the patient and the character of the disease. Their uniform effect, with I believe a solitary exception, has been very materially to relieve, and, in not a few instances, entirely to remove the cough. On several occasions, when they have not been taken every day, patients have been requested to observe whether their cough was better on those days in the morning of which they took the emetic. The answer has generally been decidedly in the affirmative; but it has certainly sometimes been, that though the cough was much lessened, it was not especially so on any particular days. But the decrease or cessation of the cough has been far from the only benefit derived from the use of emetics. Their effect in relieving dyspnœa, oppression of the chest, and load at the scrobiculus cordis, has been often surprising to the patient as well as the physician. The appetite has often improved under their use, and the whole system has appeared invigorated. Only a few days since, a patient said: 'ever since I began to take the powders, my appetite, which before was bad, has been ravenous.' Their effect, also, upon the expectoration has been very marked but various. In some cases, when previously profuse, it has been checked, and in others stopped; and while some patients have observed 'they brought up the phlegm more easily,' others, whose cough had before been dry, have experienced ease from the excited secretion of the bronchial membrane. It must however be confessed, that some have complained of the continuance of languor and nausea for a considerable part of the day, and of the exhaustion which they have temporarily endured. But even in these cases I have never yet known any permanently injurious consequences result from their frequent employment."

He admits that discrimination should be used in selecting the cases for their exhibition. As a general rule, the earlier the stage, and the more chronic the character of the disease, the greater has been the benefit derived from their operation. In many cases of incipient phthisis, they, combined with other means to be afterwards mentioned, have certainly checked and apparently removed the complaint; and in some examples of old chronic disease, accompanied with evident dulness below one or both clavicles, but without any evidence of cavities or softening tubercles, great and obvious advantage has resulted from their use. In acute or febrile phthisis, they, like every other remedy, have seemed to do little good. Where considerable debility and much perspiration have existed, the advantage derived from them has been doubtful; and when hectic has been present, and softening has decidedly commenced, they have not seemed to check the progress of the disease. Yet they have often given considerable temporary relief, and Dr. H. not only believes them the most effective remedies in the early stage of phthisis that he knows of, but that they hold out a probable hope of the cure, or, at least, the suspension of the malady.

2. *Depletion.*—Dr. H. has never ordered venesection, unless there have been *symptoms*, as hæmoptysis or pneumonia, which appeared to indicate the necessity of employing the lancet. Local bleeding has, however, been prescribed extensively and advantageously, especially in cases in which, from the physical signs, there existed evidence of bronchitis in that part affected with tubercles, or of local pneumonic consolidation resulting from their deposition. Three or four ounces of blood have been taken by cupping, or six or eight leeches have been applied below one or both clavicles; and have been repeated in three or four days, or a week, if auscultation has proved that the local inflammation has not been removed or materially reduced. The effect of this practice has been very beneficial; and the patients have loudly expressed the relief it has afforded them. Unless, however, local congestion, bronchitis, or pneumonia has been present, even topical bleeding has not been prescribed.

3. *Counter-irritation* has generally been ordered in different forms, according to the nature of the cases in which it was to be used. In those accompanied with local bronchitis, after, or in some instances without, the use of topical bleeding, small blisters have been applied below the clavicles, and repeated once or oftener, according to the effect produced. In the more chronic forms of the complaint, the tartar-emetic ointment has been preferred; while in the cases in which the patient has been delicate, and susceptible, in which there has been a more or less abundant clear serous-looking secretion from the bronchial membrane, occasionally mixed with streaks of blood—whether there has been tolerably decisive evidence of the presence of tubercles in the lungs, or no physical sign of their existence, and merely a fear of their probable deposition—or whether softening has already commenced, Dr. Hughes has ordered the liniment, composed of acetic acid and turpentine, recommended by Dr. Stokes, to be freely rubbed on the chest, night and morning. The effect of the blisters has been in the particular cases for which they have been ordered, as in ordinary cases of bronchitis unaccompanied with tubercles, almost always beneficial. The liniment—which, for the purpose of extemporaneous prescription in hospital and dispensary practice, has been composed merely of one ounce of strong acetic acid and two ounces of spirit of turpentine shaken together—has been of very great service. The advantage derived from the use of the tartar-emetic ointment, and other counter-irritants, in the chronic examples of the complaint, has been much less decided. Dr. H. doubts, indeed, whether, when ulceration has not already commenced, any positive benefit has resulted from their application.

We cannot say that this quite accords with our own observation and experience. Small leechings, occasionally employed, succeeded by the use of either blisters, the liquor lyttæ, or the tartar-emetic in ointment or in plaister, have, in our hands, been productive of much service.

4. *Antimonials* have been principally ordered in the cases accompanied with bronchitis and the expectoration of viscid frothy mucus. Under such circumstances, the tartrate of antimony, in doses of one-eighth or one-sixth of a grain, with or without a few grains of the extract of conium, has been given with good effect, but has been withheld immediately the inflammatory symptoms have ceased. In another, and in many respects an opposite class of cases, in which cough, accompanied with, and in some instances apparently dependent on, unusual dryness and irritability of the bronchial membrane, has been the symptom principally complained of, Dr. H. has prescribed the antim. oxysulphuret. in doses of from five to eight grains, to be taken three times a-day. The operation of the medicine has been uncertain. In some instances, it has produced no effect, either good or bad; while in others, very marked relief, occurring simultaneously with the appearance of the bronchial secretion, has followed its administration.

*Iodine.*—It was not unnatural to resort to it in phthisis. Let us hear what Dr. Hughes has to say of it. By him iodine and its preparations have been freely and frequently administered, and in some cases with very excellent effect; the patient's appearance having improved, and his strength increased, under their use. In others little or no benefit was derived from their employment, and the patients have gradually discontinued their attendance; while in some few, the cough has been aggravated, and the stomach has been rendered irritable by the medicine even in small doses. It has been given in doses of one-twelfth to one-eighth of a grain, with two or four grains of the iodide of potassium, and half a drachm or a drachm of syrup of poppies, either in simple water, or more commonly in the infusion of calumba. Dr. H. states his conviction, that iodine and its preparations are very valuable remedies in the treatment of incipient phthisis.

*Sedatives.*—Dr. H. has only prescribed them to quiet the cough, and occasionally procure rest. When the cough has occurred in paroxysms, hydrocyanic acid has been sometimes given with temporary benefit; though its action has certainly not been so strikingly advantageous, even under such circumstances as some Italian physicians have represented it. In other cases, a few grains of conium have been added to other important remedies; and either it, hyoscyamus, or the muriate of morphia, has occasionally been given at bed-time, to procure sleep.

*Tonics.*—When the local irritation produced by tubercles has been subdued, or when it has not existed, tonics have been freely administered, with the view and hope of improving the general health of the patient, and of changing that morbid condition of the fluids and solids of the body upon which the secretion of tubercular matter probably depends. They have usually been given in conjunction with iodine: but where this medicine has disagreed, or not acted beneficially, either the oxide of iron, or the tinct, ferri sesquichlor., has been combined with a bitter infusion: or a grain of sulphate of iron, and one or two of the sulphate of quinine, have been prescribed with infusion of roses. Together with these have been ordered as nutritious a diet as the stomach was able to digest, or bear without inconvenience; moderate exercise in the open air; and, where they were attainable, the use of horse exercise—a residence in the country, where the air was mild and dry—and a constant exposure to its invigorating influence, which Dr. Hughes regards, and we agree with him, as the best tonics of all. In fact, so far as we have seen, tonics are much abused, and often mischievous.

Dr. Hughes concludes his paper with a notice of the principal forms of phthisis, and the remedies generally useful in them.

1. *The Bronchitic.*—That variety, of by no means unfrequent occurrence, in which a person who is either hereditarily or accidentally predisposed to consumption, but who, with the exception of great susceptibility to catarrh, and the existence of more or less marked indications of tubercular cachexia, has hitherto presented none of the ordinary pulmonary symptoms of phthisis, is troubled after an ordinary cold—perhaps but little more severe than usual—with a persistent cough, with frothy mucous sputa, hurried respiration, and slight febrile excitement. On examination of the chest, there are discovered, *confined to the apex of the lungs*, a mucous rattle, which is generally distinguishable, by a delicate and well-educated ear, from the " moist crackling" of softening tubercles; an increased resonance of the voice in one or both infra-clavicular, acromial, or supra-scapular regions; and sometimes, by accurate comparison with other regions, a slight dulness or modification of sound, on percussion of the parts affected. In these cases, the use of cupping, or the application of leeches, below the clavicles, with the internal exhibition of a sixth or quarter of a grain of tartrate of antimony, sometimes combined with two or three grains of the extract of conium, and an occasional saline aperient, have been first prescribed, for the reduction of the local inflammatory symptoms. If, in a week, they have not ceased, or been materially diminished, the remedies have been repeated, or the topical bleeding has been replaced by small blisters. When the bronchitic symptoms have disappeared, as they usually have under this mode of treatment in a few days,—and when cough, hoarse, dry, or rough inspiration, with an increased duration or intensity of the expiratory murmur, and slight modification of the voice, have been left behind,—emetics every other morning, a mixture containing iodine, and the acetic acid and turpentine liniment, have been employed with very excellent effect. After the use of these for a few weeks, the patient has often presented no other general symptom of his complaint than a pale face and slightly accelerated pulse. Tonics, country air, and a nutritious diet, have then been recommended; and their use, when attainable, has been followed with great improvement of the general health, an increase of flesh and colour, and sometimes with an absolute removal of every physical sign of tubercular deposit, except perhaps a very little horseness of the respiration.

It is not always, however, that things go quite so favourably as this.

*The Hæmoptysic.*—" If a person of apparently vigorous constitution, and in good health, whose respiration has been previously unaffected, or whose dyspnœa has approached so gradually as to have escaped the observation both of the patient and his friends, has been suddenly attacked with hæmoptysis to a large amount, which, by appropriate treatment previously adopted, has been subdued, and has not since recurred; if, since the occurrence of the hæmorrhage, he has been constantly troubled with a cough, from which he had not before suffered; if the presence of pulmonary congestion has still been indicated by dyspnœa, partial turgescence of the face, a full though soft pulse, and a sibilant or sonorous rattle, mixed at some parts with soft crepitation; venesection, with saline aperients and the mineral acids, have been at first ordered, and have been followed by topical bleeding or blisters. If the hæmoptysis has recurred, or has only recently taken place, a mixture, containing the acetate of lead, acetic acid, and opium, has on some occasions been added to the means already mentioned; and, in others, a tea-spoonful of the spirit of turpentine has been ordered, to be taken at the accession of the hæmorrhage. This last remedy I have sometimes known almost immediately to succeed, when other medicines had failed in arresting the bleeding. In other examples, in which, after coughing up a small quantity of blood, the sputa have continued to be tinged with that fluid, or in which the expectoration has contained only a few streaks,—and the patient has at the same time been of lax fibre, with a small and feeble pulse, and without any febrile excitement,—I have, together with saline aperients and a blister, given eight or ten minims of tinct. ferri sesqui-chloridi three or four times a-day, with very great benefit."

Dr. H. has never known emetics either excite or reproduce the hæmorrhage. Yet he is chary of them, until hæmoptysis has passed away.

*The Simple Chronic,* which includes those cases not accompanied by either bronchitis or hæmoptysis. In this form of the complaint, emetics have generally been ordered to be taken every morning, and have often been continued for three or four weeks; the frequency of their repetition, and the continuance of their use, being regulated by the duration, the decrease, or cessation of the symptoms.

Their effect upon the cough has been really astonishing; and their influence upon other symptoms, though perhaps less obvious, has been exceedingly beneficial. In conjunction with frequently-repeated emetics, iodine, in the form previously mentioned, has been almost always administered in these cases; and the tartar-emetic ointment, as a counter-irritant, at the same time applied to the infra-clavicular regions. When the cough has entirely ceased, and most of the physical signs have disappeared, more decided tonic remedies, as the iodide or sulphate of iron, a nutritious diet, and country air, have been recommended.

A valuable paper.

---

### Observations on Diabetes: with Cases illustrative of the Efficacy of Ammonia in the Treatment of that Disease. By George H. Barlow, M.A. & L.M.

The following paper is one of a kind that the improved state of modern organic chemistry is giving rise to. Hypothetical perhaps, yet displaying theory based on science, and very superior even in its errors to the speculative spirit of the olden time.

Dr. Barlow observes that the opinion of Sydenham is rather gaining ground that diabetes is referable to a derangement of the stomach and chylopoietic viscera, rather than to a perverted action of the kidneys. This opinion he canvasses chemically and ingeniously, if not satisfactorily.

He quotes Müller for the purpose of shewing that the urinary secretions is "the means of carrying out of the system decomposed and effete animal matter, such as urea and lithic acid (the essential components of the urine), superfluous saline matters, and, either in an altered state or in their original condition, foreign matters which have accidentally entered the circulation." Dr. Barlow adds, that the urea and lithic acid, the essential components, are highly azotized combinations; whilst in the graminivorous mammalia the place of the lithic acid is supplied by hippuric acid, a substance containing little more than seven per cent. of nitrogen: so that, of the great depurating organs of the body, the office of the lungs appears to be, to separate carbon in the form of carbonic acid; the liver removes carbon, hydrogen, and nitrogen, but chiefly the former; so that it may be regarded as in great measure co-adjutive or supplementary to the lungs, but as having a superadded function of its own: the kidneys, again, separate carbon, hydrogen, and nitrogen, but principally the latter (as well as superfluous water); so that they are in some measure co-adjutive to the former, but have a superadded function of their own: the skin, again, is supplementary to all the rest, but particularly the kidney, and has an additional function of its own.

"Upon this view of the subject," he argues, "we should expect that the office of one of these organs may be wholly, or in part, performed by one or all of the others; and, accordingly, comparative anatomy teaches us, that in the different classes of vertebrate animals, the development of the liver increases in the same measure as the respiration diminishes; being at its maximum in fishes, at its minimum in mammalia, and having its mean amongst reptiles: whilst we learn, from every day's experience, that when the bile ceases to be poured forth by the ductus choledochus, it makes its appearance in the urine.

The presence, therefore, in the secretion of one organ of a substance more nearly allied in its elementary composition to that of another organ, should rather be ascribed to a defective performance of function in the latter, than a perverted action in the former; or, in other words, the presence of a highly-carbonized product in the urine would indicate impaired function of any other excreting organ rather than of the kidney. Hence the *à-priori* arguments to be drawn from the physiology of the kidney are opposed to the belief that the presence of sugar in diabetic urine is the effect of a morbid condition or action of the kidneys.

Again, what is to be inferred in reference to this subject from the pathology of other morbid conditions of the urine? or what do other morbid products indicate?

Not to encumber this communication, the object of which is more especially of a practical nature, with observations not bearing directly upon the subject before us, it will be sufficient for our purpose to remark, that of all the other morbid contents of the urine, there are none except those which are component parts of healthy blood, the presence of which has been traced to disease of the kidney; consequently, the knowledge we possess of the pathology of other diseased conditions of the urine affords no *à-priori* reason for ascribing the presence of sugar in the urine, which is never found in healthy blood, to disease of the kidney.

Since, then, there is no antecedent probability that the presence of sugar in the urine is the result of any lesion of the kidney; and since sugar has been detected in the blood of the subjects of diabetes by Mr. M'Gregor, Ambrosiani, and Dr. Rees (the latter of whom has proved that it exists in considerable quantities); it would be well to inquire how far its presence in that fluid is capable of accounting for the symptoms of this disease:—and, first, as regards the urine.

It appears, from the conclusions drawn by Woebler from his researches, 'that all soluble, and not gasiform matters, which do not suffer decomposition in the system, are got rid of by the kidneys;' whence it is evident, that sugar, being taken up into the blood, must necessarily appear in the urine. It has indeed been argued, that the quantity of sugar found in the blood of diabetic patients is not sufficient to account for the quantity in the urine. This objection can, I think, have little weight, when we consider how quickly many soluble substances, when introduced into the system, are expelled by the urine. Again, it appears that those matters which are prone to pass out of the system by any particular secreting organ are stimulants of that organ; as is seen in the stimulant action of the neutral salts on the kidneys; whence sugar, being present in the blood, and being separated from it by the kidneys, must necessarily increase the flow of urine.

There remains one other condition of the urine to be noticed; namely, the diminished quantity of urea. This deficiency is, however, by no means certain; as it has been called in question by several very able chemists; amongst others, by Mr. Kane. It may not, perhaps, be satisfactorily made out, that urea is expelled in this disease in as large quantities as in health; but I think that it has been shewn that enough is separated to confute the opinion frequently expressed, that sugar is formed instead of urea. Indeed, I should be inclined antecedently to expect that it would be deficient in this disease; since nutrition and excretion being, in health, antagonist processes, it is not improbable that the diminution of the former by disease may induce a deficiency of the latter.

It appears, then, that the presence of sugar in the blood is of itself sufficient to account for the abnormal condition of the urine."

The thirst and dryness of the skin may be ascribed to the drain of water from the diuretic action of the sugar—the hunger to the atrophy, the consequence of the depraved state of the blood. Then which is the erring organ? Dr. Barlow reasons thus:—

"The circumstance of the morbid ingredient being a highly carbonized substance might at first lead to the belief that either the lungs or liver, or both, were involved in its causation; and it is probable that they are more or less implicated: but the fact, that sugar has been discovered by Mr. M'Gregor in the stomachs of diabetic patients in greater quantities than in health, even when such patients have been almost entirely restricted to an animal diet, affords a proof that the derangement in sanguification must take place when the nutrient fluid is in an earlier stage than that in which it is subject to the action of these organs; and that the primary disturbance may be traced to the primæ viæ. And here it would be well to reflect upon the product of digestion in health, and in this disease. I again quote the words of Müller :—' The end and object of digestion is, first, the solution of the food, since nothing can be taken up by the absorbent vessels which is not in solution ; and, secondly, the reduction of the different ingredients into the most simple material of the animal processes, namely albumen, which is found to be contained in the fluid resulting from the digestion of the food, partly in the state of solution, and partly in globules. The essential character of the digestive process consists in its not only effecting the solution of the food, but in its likewise annulling the peculiar properties which the nutritive matters may owe to the source whence they are derived ; that is to say, in dissolving the food, and converting all into albumen.'

In this disease, on the contrary, the saccharine particles of the food are not changed in the stomach; whilst the starch, which most articles of vegetable diet contain in considerable quantities, not having its peculiar properties annulled, and its proneness to the saccharine fermentation being favoured by the warmth and moisture of the stomach, is converted into sugar, which, being readily soluble, is absorbed into the circulation."

Thus, from some defect in the assimilating power, a lower product, starch, is found in lieu of a higher one, albumen. The former is inadequate to the purposes of the economy, and is eliminated by the kidney. It is not, however, actually proved that there is a diminished quantity of albumen in the blood, though that is probably the case. Let us turn to the remedial principles founded on the preceding views.

"The next inquiry is, what are the inferences to be drawn from it respecting the treatment of diabetes. The first is obviously one, the correctness of which has long been acknowledged, and confirmed by experience; namely, the avoidance of all saccharine and amylaceous articles of food; the latter of which, from their tendency to saccharine fermentation, are, I believe, productive of as much mischief as the former. I have, however, little to add to what is generally received upon this point, further than to insist on the advantages of the cruciferous vegetables as articles of diet; the use of which is not only in accordance with the view taken above, but has received the sanction of many experienced physicians. Not only do greens, broccoli, turnip-tops, sea-kale, water-cresses, &c. tend to obviate the loathing which is often felt by these patients when restricted to an animal diet, but they exert a decidedly beneficial influence over some of the symptoms : and it will be seen, in a case to be related presently, the discontinuance of the use of greens was always followed by increase in the flow of urine.

The next indication appears to be, to introduce into the stomach a highly azotized substance, and at the same time, by a diffusible stimulant, to exalt, if possible, the assimilating powers of that organ ; both which ends appear likely to be attained by ammonia.

There is one circumstance connected with the employment of ammonia in this disease ; upon which, however, I do not wish to lay great stress, although it is at least a coincidence too remarkable to be passed unnoticed ;—I mean the chemical relation of sugar, ammonia, and albumen, as regards their elementary composition. Thus we find, that when the numbers which represent the atomic composition of ammonia and sugar are added in certain proportions, we obtain a result which exactly coincides with the numbers representing the atomic composition of albumen, increased by certain equivalents of carbonic acid and water, substances which are continually excreted from the body.

| | Carbon. | Hydrogen. | Oxygen. | Nitrogen. |
|---|---|---|---|---|
| 9 Eqnts. Sugar ........ | 55.08 .... | 9 .... | 72 .... | 0 |
| + 1.1 Eqnt. Ammonia...... | .... | 3.3 .... | .... | 15.56 |
| | 55.08 .... | 12.3 .... | 72 .... | 15.56 |
| — 5 Eqnts. Water ........ | .... | 5 .... | 40 .... | |
| — ½ Eqnt. Carbonic Acid.. | 3 .... | .... | 8 .... | |
| | 52.08 .... | 7.3 .... | 24 .... | 15.56 |
| Which, reduced to 100 parts, gives .... | 52.63 .... | 7.37 .... | 24.25 .... | 15.73 |
| Albumen (according to Gay Lusac & Thenard) | 52.88 .... | 7.54 .... | 23.87 .... | 15.70" |

Dr. B. has found that under the use of the sesqui-carbonate of ammonia the function of the skin is generally restored; though he has sometimes thought that opium assisted in effecting this result. Such exercise as the strength of the patient will enable him to take, and the use of a warm bath occasionally, where it can be obtained, are also valuable adjuvants. The accumulations which not unfrequently take place in the large intestines are best removed, he thinks, by a purgative with a tonic—rhubarb and sulphate of potass, aided, if requisite, by castor oil, are preferred by him.

Dr. Barlow relates five cases. We shall select the first as a sample, and perhaps a favourable one of the whole. For the remainder are unfortunately far from conclusive as to cure, though conclusive enough as to benefit. They resemble many other cases, treated by many other remedies, that have been from time to time brought forward with the flattering anticipation of more decided and permanent advantage than has been obtained. We fear that we can hardly dare to look to a diffusible stimulant, as a means of a class that *must* have been often tried by doctor and patient, for victory over so intractable a malady. But to the case.

*Case* 1.— —— Stanley, a shoemaker, applied to me, at the Surrey Dispensary, in the summer of 1836; stating that he had been told that he was the subject of consumption, having lost flesh and strength very rapidly for about a month. I accordingly examined his chest; but could detect none of the physical signs of phthisis. Upon further inquiry, I learned that he had for some days been struck with the great increase in the quantity of his urine, which amounted to fifteen pints in the twenty-four hours. He was, moreover, much emaciated: his skin was very harsh and dry, which he told me was the case by night as well as by day: his tongue was loaded: he complained of great thirst; and said that his appetite was excessive. His urine was sweet to the taste, and of sp. gr. 1.041. He was at first put upon the use of one grain of opium every four hours; some castor-oil being ordered occasionally, to regulate the bowels: the rules respecting diet, above laid down, being also enjoined.—At the end of five days his urine was much diminished in quantity; but the specific gravity increased to 1.044: he was also at that time in a state of great prostration, which was perhaps in some measure attributable to the opium. He was then ordered to take six grains of the sesquicarbonate of ammonia three times a day, in a draught containing one drachm of sp. lavend. comp.; and five grains of Dover's powder every night; by continuing which, the quantity of the urine was, at the end of eight days, reduced to twelve pints in twenty-four hours, and the specific gravity had decreased to 1.035: he also perspired moderately at night, and the thirst was much abated. I now increased the quantity of the ammonia to eight grains every four hours; under the use of which, the quantity and specific gravity of his urine rapidly diminished; and at the end of six weeks he had so far recovered his flesh and strength, that he considered himself well, and left the Dispensary. At the end of five weeks, however, he again presented himself; stating that his former symptoms had returned. His skin was then harsh and dry; and he told me that his urine rather exceeded two gallons in twenty-four hours. He had lost flesh considerably since I had last seen him; but he was not so much emaciated as when he first came under my care: his tongue was clean, but moist, and his breath had the odour of hay. He was treated in the same manner as before; and at the end of two months he was again discharged, apparently in good health; his urine being about three pints in quantity, without saccharine taste, and of specific gravity 1.020.—I ascertained that this man was in good health in the summer of 1839."

Dr. Barlow has our thanks for his Paper. It deserves consideration.

### Operation for the radical Cure of a Reducible Inguinal Hernia. By Bransby B. Cooper, F.R.S. &c. &c.

The following case is very interesting.

A man, aged 22, tall and muscular, had had a reducible inguinal hernia on the right side for seven years, when, on the 26th of May, 1840, a larger portion was forced down, and was, with difficulty, returned in Guy's Hospital. On the slightest exertion, however, the hernia descended again. The man was employed on the Greenwich Railway, and severe exertions were necessary. These were out of the question with such a hernia, which no truss could be got to keep up. Mr. B. Cooper accordingly deemed it a fair case for the operation recommended by M. Gerdy.

On the 10th of June, the patient was brought into the operating theatre, and laid upon a table, on his back, with his chest and thighs raised, for the purpose of relaxing the abdominal muscles. Mr. Cooper then commenced the operation by pushing a portion of scrotal integument before the forefinger of his left hand, through the external ring, into the inguinal canal, as high as he could pass it; and upon the finger he then introduced a director. A long needle, fixed in a wooden handle, and having the eye, near its point, armed with a double silk ligature, was then carried along the director to the very extremity of the invaginated skin, and was pushed through the tendon of the external abdominal oblique muscle and the skin, so as to make its appearance an inch and a half above Poupart's ligament: one end of the silk was then retained by an assistant, and the needle drawn back again into the inguinal canal, along the other end; when it was again pushed through the abdominal parietes, in a similar manner as before, about four lines distant from the other end of the thread, including necessarily so much of the skin between the two silks; which were now tied over a piece of bougie, so as to retain the invaginated portion of skin within the inguinal canal. A piece of lint, wrapped around a director, and dipped into liquor ammoniæ, was passed into the cul-de-sac of skin thus formed; and the surface well rubbed with it, in order to remove the cuticle, and promote an inflammation in the cutis, so as to obliterate this integumentary canal, and to form a plug sufficiently firm to prevent the future descent of the hernia.

The application of the ammonia caused intense pain.—The patient was now taken to his bed, placed in the same position as before the operation, and the scrotum well supported. An hour and a half after the operation, as great pain was still complained of, a grain of opium was given, which afforded but little relief. At nine in the evening, Mr. Cooper saw him; at which time there was great distress of countenance, profuse perspiration, and the pulse quick, but compressible; and there was not any pain produced by pressure on the abdomen.

We need not pursue the diurnal details. The symptoms of irritation gradually passed away. A discharge, of purulent character, from the invaginated skin made its appearance on the 12th. On the 13th, the scrotum was strapped up, so as to press against the margin of the opening from the invaginated portion of skin.

14th.—The ligature was removed, as purulent discharge was now most freely established: but the pressure on the part was desired to be continued, and every thing seemed to be going on favourably. But on the next day there appeared a degree of fulness about the margin of the opening, as if a portion of the inverted skin had descended; but without any descent of intestine, and the hardness and swelling about the inguinal canal still led to the reasonable hope that the operation would prove successful. In this state he continued for several days, until the tenderness about the inguinal canal had sufficiently subsided to allow of a greater degree of pressure being made upon the part; the patient being still kept in the recumbent posture.—After that time all uneasiness had left him; and the patient described that he felt the affected side as firm as the other; which the thickening in the course of the canal seemed to justify. On the 4th of July, a weak truss was adjusted to the part; and he was still desired to remain in bed; which he did for ten days more, when they could no longer keep him in bed. He remained yet a fortnight in the hospital, wearing his truss, and walking about without any descent of the hernia occurring; and left Guy's at the end of July, to resume his occupation on the rail-road, with the promise of being, at first, put only to slight work. This was observed, and early in August, he resumed his old occupation as hammer-man, a more severe one. Unfortunately, he was only protected by the weak truss which was given to him while in the hospital: so that, upon one occasion, a portion of intestine again descended into the inguinal canal, while at work. A much stronger truss was then substituted for the weak one; and since its application, he has had no return of his complaint, but is enabled to perform all the duties of his situation.

Mr. Cooper observes:—It is true, from the history of this case, that the operation has not entirely succeeded, not having led to the perfect obliteration of the inguinal canal: but still it is to be remembered, that, before he submitted to it, no truss could prevent the descent of his hernia rendering him entirely incapable of the slightest exertion; while now, on the contrary, by the use of a common truss he is rendered an efficient labourer: and there is little doubt, had a proper truss been employed before he resumed his more laborious occupation, that no protrusion would have ever recurred; and that in a year he might have left off the use of a truss altogether; which, under the present circumstances, it will not be safe for him to attempt.

### OBSERVATIONS ON ABDOMINAL TUMORS AND INTUMESCENCE: ILLUSTRATED BY CASES OF DISEASED LIVER. By R. BRIGHT, M.D. F.R.S. &c.

In following out the subject of abdominal tumors, Dr. Bright draws, in the present Paper, his illustrations from the *Liver*.

In seeking, says our author, to render our diagnosis as correct as possible, in any case of hepatic disease, we are necessarily led to attempt to discover the size and form of the affected organ; a task, in many cases difficult, if not impossible; and sometimes, when performed, liable to lead us astray, unless we carefully take many other circumstances into consideration; but at other times affording us the most important information towards the discovery of disease.

One of the chief sources of difficulty, in ascertaining the size and form of the liver, depends upon the situation of the organ: for it is so placed, with regard to the ribs and the diaphragm, that, in its most perfect state of health, it is almost as much concealed from the sight, and removed from the touch, as the contents of the cranium. Another difficulty arises from the liver being liable to displacement, from causes independent of disease within itself; as from occasional, though not very frequent deviations from its natural position, and from pressure exerted upon it by effusions within the right cavity of the chest, or from tumors between the liver and the diaphragm. And a third source of difficulty is found in the induration and enlargement of neighbouring organs; as of the right kidney, the stomach, the omentum, and the colon. Still in most cases we can arrive at a very satisfactory knowledge of the size and form of the liver, when it deviates at all considerably from its normal state.

Dr. Bright observes, in reference to the natural site of the viscus,—The large right lobe of the liver, in its healthy state, lies completely in the hollow formed by the diaphragm, not descending below the margin of the ribs, and extending upwards to between the sixth and seventh ribs on the right side. The left lobe usually extends to the soft space below the ensiform cartilage, a short way into the left hypochondriac region; and a portion of its lower margin is thus seen lying across the scrobiculus cordis when the body is opened, and is frequently the only part of the organ which is visible.

As a necessary consequence, he goes on to remark, of this situation, the healthy liver influences very little the sound produced by percussion on the soft part of the abdomen; which, if all the organs are free, healthy, and empty, is usually clear and sonorous, from immediately below the margin of the ribs, to the very lowest part of the pubic region. If, then, the sound in any part be dull, it is our business to ascertain the extent and connection of such unnatural sound: and in this way, if we can trace an uninterrupted dulness to the margin of the ribs on the right side, our suspicions may fairly be excited, and the liver is the origin of the disease. The more perfect and the more practised the ear, the more likelihood there is of tracing the deviations of sound from their natural clearness: but in some cases of very extensive disease, where the liver or other organs are irregularly enlarged or tuberculated, the investigation is most difficult. Yet still, Dr. Bright thinks the touch more fallible than the ear, in cases of extensive tubercular or fungoid deposit in the abdomen. We must never suffer ourselves to be led into the error of denying the existence of hepatic tumor because the dulness or the hardness are so extensive that they appear to reach beyond the probable bounds of the liver; for, in fact, there is no tumor, of which the abdomen will admit, so large that it may not be an enlarged liver: and if we can satisfactorily trace the continuity of the dull sound, or the hardness under the ribs of the right side, while no other obvious indication leads us to ascribe it to another organ, we may legitimately consider the liver as the seat of the disease. But after all, we shall not be always right.

It would be an error to suppose the rapidity with which the tumor has appeared to be inconsistent with the idea that it can have originated from the liver; for we find tumors of the most extraordinary extent generated in the liver in a few weeks:—nor are these always attended with such remarkable pain as might be expected under such rapid distention of structure.

The general symptoms or condition afford valuable assistance.

Disease of the liver seldom exists long without producing a peculiar appearance in the countenance of the patient. In some cases, as we shall see, actual jaundice, and that of the most decided character, accompanies hepatic tumors; but many of the more formidable conditions of the liver are indeed, but slightly marked by this symptom. Still, the approach to the jaundiced state, the sallow cheek and temples, and the lightly-tinged conjunctiva, are most often present when disease has greatly altered the structure of the liver, or gone on to the formation of tumor. To this, however, there are remarkable exceptions; so that the absence of the symptoms should never lead us to repudiate the idea of hepatic disease. Fungoid growths to a very considerable extent may occupy the liver, and yet no jaundice, and no approach to it, may be present. Fatty intumescence of the liver has often been recognised by a peculiar marbly appearance of the skin that it gives birth to.

2. Gradual or rapid emaciation, with a peculiar cachectic aspect, frequently accompanies disease of the liver; though even this is far from constant; for there are certain forms of disease in which the liver is enlarged, and which are marked rather by an increased deposit of fat in the cellular membrane of the body, and in the omentum and mesentery, than by emaciation. The state of the bowels and the stomach greatly assists our diagnosis. Hæmorrhages taking place from the stomach and intestinal canal, and effusion of serum into the cavity of the abdomen, are amongst the symptoms which call our attention to the condition of the liver, and often strengthen our diagnosis.

The tumors depending upon the liver vary greatly in the extent they occupy, as also in their characters; sometimes descending scarcely below the margin of the ribs, and sometimes encroaching upon the pelvis. They are sometimes smooth and even; sometimes lobulated, with greater or smaller inequalities on the surface; sometimes soft and yielding; sometimes hard.

Dr. Bright, before entering on the consideration of hepatic tumors themselves, points out the growths or morbid affections most apt to be confounded with them.

1. *Accumulations in the Colon.*—Amongst, says Dr. Bright, the many sources of such mistakes, by which physicians may be misled, and induced to conclude that the liver is the seat of disease when in fact it is not, feculent accumulations in the colon are perhaps the most frequent; and they lead to a deception the more complete, because they occasionally imitate, in the most striking manner, enlargements of this and other organs, and appear to afford a decided and tangible evidence of disease such as few can withstand, even to afford time for making trial of remedies, which, by acting freely on the bowels, might at once shew the cause, and remove the tumor. Dr. Bright relates the particulars of four which have occurred under his own observation.

The *first* case was one of accumulation of fæces in the sigmoid flexure of the colon, imitating organic tumor.

The *second* case was one of fæcal accumulation in the colon, imitating hepatic enlargement.

The *third* case—fæcal accumulation in the colon, imitating fungoid tumor.

The *fourth* case—fæcal accumulation in the colon, imitating malignant disease of the liver.

We must content ourselves with quoting the fourth case, one as much in point as any.

"CASE 4.—*Fæcal Accumulation in the Colon, imitating Malignant Disease of the Liver.*

A. B., a seafaring man, aged about 55, was admitted into Guy's Hospital, under my care, with a hard lobulated tumor, about midway between the point of the ensiform cartilage and the umbilicus, in which he suffered considerable pain, both from pressure and without it. His complexion was sallow: his bowels stated to be freely opened. After careful examination, I had very little doubt that the tumor was organic, and connected with the left lobe of the liver: nor did the effect of remedies, or the appearance of the patient, at all undeceive me for some weeks; but I presently began to suspect that the pains, of which he made such frequent complaint, were rather of a spasmodic character, and such as indicated some detention of fæces in the intestine. I therefore put him on a more decided plan of purging than at first, though the bowels had never been neglected. He now took repeated doses of compound extract of colocynth, galbanum pill, blue pill, and small quantities of muriate of morphia. The effect was, after a few days, to bring away a quantity of hardened balls of fæces; and, in proportion, to diminish the supposed malignant tumor, till both pain and morbid growth, and every other symptom of disease, had disappeared."

Dr. Bright would be inclined to say, that, whenever an abdominal tumor occurs, in what may be considered the course of the colon, we should be very guarded in our diagnosis: and yet this will hardly cover all the possible cases of deception; for the colon is itself, of all the viscera of the abdomen, that which varies most in its course; so that scarcely a month passes in which we have not an opportunity of witnessing some variation:—as an illustration of which, he refers to three instances which he saw within ten days of each other. In one, the arch of the colon suddenly descended below the umbilicus; in another, the sigmoid flexure advanced beyond the same point; and in the third, the sigmoid flexure performed two complete convolutions, the least of which ascended to the duodenum where it commences in the stomach, and then descended to the pelvis.

2. *Disease of the Kidney,* frequently occasions difficulty in diagnosis. For though it seldom enlarges in such a way as to push the right lobe of the liver before it, yet it often presents itself as a tumor, proceeding from the under surface of the right lobe: and as it has sometimes attained a considerable size before it has been detected, it has been supposed to be continuous with the liver, and a growth from its substance.

3. *Disease of the Stomach.*—Disease of the stomach might be mistaken for tumor of the liver, particularly of the left lobe; but this will not often occur. The small curvature, when scirrhous, and particularly when fixed by disease to the liver, resembles greatly hepatic tumor. A malignant tuber in the stomach likewise, or a malignant thickening of the whole of that organ, may at first sight deceive; but strict examination, particularly by percussion, will demonstrate the cavity beneath, and shew that the disease is situated in a hollow viscus. In general, the pain referred to the stomach, and increased or excited by eating, the frequent nausea or vomiting, the marked emaciation, and the absence of the more remarkable symptoms of hepatic disease, will enable us to determine that the tumor belongs rather to the stomach than the liver.

4. *Morbid Growths of the Omentum or Peritoneum* may assume a very near resemblance to the liver studded with tubera or enlarged by disease: in most cases there will be found an obvious separation between the tumor and the liver, and a space where the colon or the stomach emits a clear sound on percussion; and the hard portions in the enlarged abdomen will be separated in a manner which will prove that they are not connected with the liver: there will likewise be an absence of many of the symptoms of hepatic disease. Dr. Bright, however, introduces one case for the purpose of shewing the difficulties that may be experienced.

Case.—*Malignant Disease of the Peritoneum, resembling Hepatic Tumor.*—On the 22d of November, 1830, Dr. B. was requested by Mr. Fernandez, to see a shoemaker, aged 44. The account obtained was, that about a year ago he first felt a small lump below the ensiform cartilage; and the hardness seemed to increase across the stomach at the upper part, gradually extending downwards, to the present state: for some months he had occasionally vomited his food; and for the last six weeks this had happened constantly, about half-an-hour after eating, without pain or difficulty: though the nurse said that what he vomited was of a dark colour, having both the appearance and smell of fæcal matter. The stools were dark. His countenance pallid, but not sallow; and he had never had any thing approaching to jaundice. On examining the abdomen, there were two or three projecting lumps, of the size and nearly the shape of half an egg, near to the scrobiculus cordis; and the whole upper part of the abdomen presented one uniform hard substance, almost as firm as cartilage, giving a general and equal fulness to the abdomen: this hardened condition extended almost to the pelvis, where there was a distinct lobulated margin to be traced, in the form of the lower margin of the liver: this descended lowest on the right side, but also was low on the left, where one or two lumps were to be felt, like independent tubers separated from the general mass. Increasing exhaustion took place, and, on the 9th of December, the man died. On the 11th the body was examined.

On removing the external integuments and muscles, the peritoneum remained thickened to nearly a quarter of an inch, in some parts: and when this was thrown back, a large mass, very firm, and nearly the colour of fat, presented itself, descending into the pelvis, and there assuming the form of the liver, with a division between its lobes. This mass extended upwards, so as to push the diaphragm before it, and assume nearly the form of that muscle, in expiration. Raising this mass from below, the intestines came into sight, pushed chiefly to the left side, and covered with rounded masses of a semi-gelatinous form and appearance, assuming quite the disposition of fungoid disease: sprouting up, and growing in botryoidal forms, and giving an indistinct vesicular appearance, when cut through. Many of these fungous masses were arranged near the point where the mesentery joins the intestine; and some were quite pendent by threads not less than half an inch; and some had three or four such threads supporting them, apparently vessels. Many were seated upon the mesentery, or on the intestine. A large mass had formed between the rectum and bladder.

On examining more carefully the large mass which filled the greater part of the abdomen, it was found to be almost entirely formed of the adventitious structure, and the liver and stomach were both included in its substance; the liver not greatly altered in its colour or texture, but dwindled in size; and the stomach greatly contracted, and rendered quite irregular through its whole internal surface, so that the cavity bore no resemblance to the natural form or appearance. This mass likewise descended to the kidneys, which were partially imbedded in it. It could be raised from its lower margin like an enlarged liver, and then the intestines were displayed; but the fungous granulations from the different parts had produced some adhesion. The texture of this mass was quite vesicular; and though it seemed formed of numerous cysts, of almost equal size, not larger than sweet-peas, so that great part of it presented a rather uniform texture, it was evident that it assumed, in some of its loose and less-restrained portions, the structure which Dr. Hodgkin has ascribed to malignant growths; and in many of the cavities a gelatinous matter had collected, as in the ovarian dropsy.

The lungs were healthy, but there were several little transparent fungoid bodies on the pleura. The peritoneum covering the liver was greatly thickened by the same morbid growth, and adhered to the viscus.

4. *Displacement of the Liver by Disease in the right side of the Chest.*—It frequently happens that extensive effusion, or consolidation of the lung, either from pneumonia or malignant disease, depresses the liver so much as to render the sound of the right hypochondrium most remarkably dull for several inches below the ribs; and then it is by no means uncommon to find the medical attendant fully convinced that the liver is enlarged;—and probably now, if not before, he is induced to doubt whether the previous inflammatory attack did not belong to the liver, rather than to the lung or pleura.

Dr. Bright relates two cases illustrative of this remark. They present, however, no feature of particular importance.

In the next case, the liver was pushed down by effusion between it and the diaphragm.

CASE.—*Abscess situated between the Diaphragm and theLiver, producing apparent Enlargement of the Liver.*

August 6, 1834.—A boy was admitted into Luke's Ward, under the care of Dr. Back, labouring under bronchitis. He became rather suddenly the subject of a very large swelling in the situation of the right lobe of the liver, but passing over, in a cushion-like tumor, towards the left side. No hepatic symptoms presented themselves. It was leeched, and other remedies employed; and at a time when it seemed to threaten great mischief, it rather suddenly diminished to a great extent: and then it very naturally became a question, whether this had been a highly-congested state of the liver from the bronchitis, or whether it might have been fæces in the colon, or whether some abscess in the liver had found means to discharge itself. The relief obtained was very temporary; and on the 11th of August he died.

*Sectio Cadaveris.*—The lungs bore decided marks of bronchitis and of pneumonia: the right lung was adherent to the diaphragm. A large abscess was situated between the diaphragm and the liver, pressing down the latter. Its parietes completely insulated it from the general peritoneal cavity; but it had so compressed the right lobe of the liver, as to produce the complete appearance of an excavation in that organ, as an empyema seems to scoop out the lung with which it lies in contact. The surface of the liver, however, was not broken; so that there was no trace of bile in the abscess.

5. *Malposition of the Liver, &c.*—"The deviations," says Dr. Bright, "from the natural position of the liver, with which I have met, have been very few; but where they do occur, they must necessarily present difficulties in diagnosis, scarcely to be overcome. I have never been present at the examination of a body in which the organ was transposed; but I have seen the left lobe so much elongated and enlarged, without any disease in the structure, as to vie with the right in size; and in other cases, to extend across to the left hypochondrium, reaching quite to the spleen. I have also seen, in one case, the liver placed behind several coils of intestine: so that whatever had been its size or extent, percussion would have yielded a clear sound."

CASE.—*Small Intestines situated anteriorly to the Liver.*

Mr. Bushfield aged 50, with sallow complexion, had consulted Dr. Bright frequently, in the last two years, for a loathing of food, and a sense of sickness of stomach without vomiting. Bowels costive, but he suffered much from the action of purgatives. He spoke of pain at the scrobiculus cordis, running back to the spine, and up the centre of the chest. He obviously became emaciated; and his symptoms were altogether such, that Dr. B. suspected some malignant disease. In the last year of his life, he had plainly phthisis pulmonalis.

In March, 1835, he died, and the body was examined. In the apex of each lung were old phthisical cavities, and some tubercles in other parts. The heart healthy, but small and flaccid. Towards the pyloric extremity of the stomach were two or three small round ulcers. Pylorus healthy: duodenum granulated. There were several ulcers in the ileum, particularly near the valve, and also in the colon. The mesenteric glands, near the ulcers, slightly enlarged: liver healthy: pancreas congested: spleen twice its natural size: kidneys healthy, but discoloured by congestion.

Such was the condition of the several organs; but the most remarkable circumstance was, the relative position of the abdominal viscera, when the abdomen was laid open. Neither the liver nor the colon presented itself to view; but, in their stead, the convolutions of the small intestines, which were found to have come completely in front of the liver; the colon and the omentum doubling over the liver, and pressing it back, so as to have made deep furrows in its anterior surface.

6. *Disease of the left Lobe of the Liver.*—Dr. Bright has seen great difficulty arise when the left lobe of the liver alone has been involved in disease, or where the disease of that lobe has been greatly disproportioned to the disease of the right lobe: of which the following case furnishes a good example.

CASE.—*Malignant Tumor, confined entirely to the Left Lobe of the Liver, and ascending towards the Thorax.*

Ann Cook, aged 59, a widow, admitted into Guy's Hospital, Nov. 6, 1839. Six weeks back, she had been wet through; and was attacked with rigors, flushes of heat and great thirst, with pain in the left side, which she says had existed in a less degree for some time before. This pain was aggravated by cough and deep inspiration.

At the time of her admission, her countenance was expressive of much suffering; and she complained of great pain in the left side, near the angle of the ribs. In the left hypochondriac region, just below the margin of the false ribs, there was a tumor of the size of a large fist, very tender on pressure, and protruding in a very obvious degree, the lower ribs. Tongue brown and dry: urine passed in moderate quantities, depositing the purpurates, and not coagulable by heat.

The right side of the chest, anteriorly, yielded a clear sound on percussion, and the respiration was natural. On the left side, anteriorly, it was dull on percussion, as high up as between the second and third ribs, and no respiration was heard: posteriorly, it was dull on percussion, the respiration tubular, and there was bronchophony. The sounds of the heart heard more to the right of the sternum.—She died in ten days.

*Dissection.*—On opening the chest, the diaphragm was noticed to be pushed up by the liver, as far as the third rib on the left side; but on the right, only as far as the sixth. The left lung was pushed up very high, as far as the seventh rib; but there was a small portion which was situated lower down, posteriorly, and which appeared much compressed: they were otherwise healthy.

The heart was pushed up, and more to the right side than natural.

Abdomen.—On opening this cavity, a large tumor presented itself: this was situated in the left hypochondriac region, and originated within the left lobe of the liver, which pushed the stomach to the right side. The tumor within the liver was of the size of an adult's head, and of a rounded form: its external surface was firmly adherent to portions of the lower surface of the diaphragm, and posteriorly to the spleen and kidney. On cutting into the tumor, it was found to be of a fungoid nature (fungus hæmatodes), originating within the structure of the left lobe of the liver internally.

7. *Other Sources of Difficulty.*—"Besides the difficulties which have already been enumerated, as opposed to unerring diagnosis, we must not omit to mention, that the spleen, when diseased, has occasionally been mistaken for the liver, and the liver for the spleen;—errors into which we may easily fall, when the left lobe of the liver is particularly affected, or is supposed to be so: nor is it an unusual thing to find both liver and spleen enlarged at the same time. It must likewise not be forgotten, that ovarian tumors, encroaching in their progress upon the right hypochondrium, and on the upper portions of the abdomen, have not only by careless and ignorant men, but by the skilful, been pronounced hepatic."

ENLARGEMENTS OF THE LIVER ITSELF.

To these, Dr. Bright now proceeds. He adopts the division into the smooth and the irregular forms of tumor.

In the first of these diseases may be included enlargement from the passive congestion of blood—from acute or sub-acute inflammation; from retention of bile; from chronic hypertrophy; from fatty changes with intumescence; and from diffused malignant disease.

In the second division—tumor of irregular form—may be included, abscess, both acute and chronic; hydatids; the result of chronic inflammation, producing irregular contractions in the cellular membrane of the liver and permanent roughness of its surface; malignant disease in the several varieties of the scirrhous, cerebriform, and melanotic deposits.

1. *Smooth Tumor or Tumefaction of the Liver from Sanguineous Congestion.*—"The most simple form," says Dr. Bright, "of hepatic enlargement is that which results from sanguineous congestion, where the increase in size is entirely owing to the unnaturally distended condition of the blood-vessels. This form of disease is by no means unfrequent in its less aggravated degree, apparently connected with loaded bowels making pressure upon the returning veins; and probably, with the deficiency and sluggishness of the peristaltic action of the intestines, encouraging delay in the circulation of the blood; which again, when once collected in the liver, proves an additional impediment to the onward progress of the stream. When the liver is thus loaded with blood, it gives rise to many of those ailments which are variously denominated dyspeptic or hypochondriacal, interfering with the digestion, and oppressing the nervous energies of the whole system, and sometimes mechanically impeding the action both of the heart and lungs.—A slight fulness is perceptible on the right side, and the ribs are a little raised. To the hand, the space below the ribs is more resisting, and even hard; and although there is no defined tumor, the edge of which admits of being traced, the dull sound which is elicited by percussion an inch or two below the margin of the ribs contrasts strongly with the clear sound of the hollow viscera which ought to occupy that space.

The enlargement from sanguineous congestion in the limited degree of which I have spoken, may be difficult to ascertain; but there is a degree of congestion betraying itself most manifestly by the enlargement of the organ, which descends several inches below the ribs, and may be felt as a hard full cushion with a defined margin, sometimes on a level with, and sometimes below the umbilicus. In cases of this kind, besides the defined character of the tumor, we have usually a peculiar sallowness of the complexion, which more especially directs our attention to the liver; and that sometimes to such a degree, that experienced physicians have been led away entirely from the primary disease on which the hepatic congestion depended, which is generally some obstruction of the circulation in the heart: and I have known, in this way, a patient supposed to sink under hepatic disease, while ossified valves, and enlarged and distended heart, have been the true cause of all the symptoms. In such cases, it is true that the liver, from being simply gorged, becomes gradually disorganized, passing from the nutmeg liver of distention to the permanent yellow and red liver, in which probably some adventitious deposit or some permanent change of character has taken place; but this is most decidedly a consequence of previous appreciable disease in another organ."

We would point attention to the latter observation, as practical as true. It was only the other day that we witnessed a case of this description. The liver was much enlarged from congestion, and had diverted attention altogether from the condition of the heart which was greatly dilated, with attenuation of its parietes, and adherent pericardium.

Dr. Bright relates a case of *Liver enlarged, and altered in its Structure from frequent congestion,* which, however, we need not go into.

2. *Intumescence of the Liver from Inflammation.*—" It is to be presumed," says our able author, " that in most cases of inflammatory action the bulk of the liver is more or less augmented, in the early stages at least. But it often happens, that the evidence of inflammatory action exists in the pulse, the skin, the tongue, and the altered secretions both from the bowels and the kidneys; and yet no very decided fulness is perceptible in the right hypochondrium : but more frequently we find, on passing the flat hand gently over the part, that it experiences a little more resistance, and a little more sense of fulness, as it arrives at the right side : and, on careful examination with the points of the fingers, we discover the margin of the liver descending from one to two inches below the cartilages of the ribs ; and, on applying percussion, the sound is dull over a corresponding space. Sometimes the part is so tender, that these investigations can scarcely be borne ; while, at other times, the patient complains little at the moment pressure is made, but suffers considerably from aching pain in the part for some time afterwards. The tumor thus produced is somewhat resisting, but not indurated ; and it gradually subsides, as the general symptoms of inflammation are subdued. Leeches and the assiduous application of poultices, are the local remedies indicated : while bleeding from the arm, mercury with or without opiates, and antimonials, together with free action on the bowels, are the constitutional remedies, which can scarcely be safely dispensed with, where so important an organ, and one so apt to run into suppuration, is inflamed."

Dr. Bright relates no cases of this form of hepatic enlargement. He thinks it more frequent where hepatic inflammation tends to suppuration and the formation of abscess, than when it leads to simple jaundice.

3. *Intumescence of the Liver from accumulation of Bile.*—" A third form of smooth enlargement of the liver is produced by the bile being retained, so that it accumulates in the biliary ducts. In such cases, the liver gradually enlarges ; and may be felt as a tense smooth tumor, descending toward the umbilicus, and proceeding onwards almost to the pelvis, while it nearly fills the right lumbar space. Pressure is productive of some pain, which often lasts for many minutes. In such cases, we are usually directed in our diagnosis by the very decidedly yellow suffusion of the skin ; and, in many cases, by a peculiar rounded tumor projecting from the lower margin of the liver. This will, however, depend upon the cause of the detention of bile in the liver. I believe that it very rarely, or perhaps never happens, that the liver is greatly gorged with its own secretion, unless some decided mechanical obstruction exists. When sanguineous distention takes place to a considerable degree, the bile is certainly more or less retained in the small tubes, and produces a jaundiced tinge on the skin : but here the obstruction is only partial, and is not fixed ; and the degree of bilious congestion, compared with the sanguineous, is but small.

The circumstances under which I have seen the liver decidedly loaded with bile to distention, so that the bulk of the organ has been enlarged, and manifest swelling produced, have been tumors, or morbid deposits, pressing on the large excretory ducts, or biliary concretions impacted within them. If the obstruction thus produced occur in the hepatic duct, the tumor of the liver takes place, and the organ is distinctly to be traced gradually descending from the margin of the ribs, towards the pubic and the iliac regions, presenting a smooth and even surface. The whole, dull on percussion ; and this dulness ascending to the sixth and fifth ribs of the right side. If the obstruction be lower down, occupying the common duct, the same enlargement of the liver takes place ; but gradually we perceive the margin of the liver deviating from its even line, and a globular projection protruding itself downwards, of the size of a small egg. This projecting portion of the tumor yields, on pressure, the elastic feel of a deep-seated fluid: it increases, and becomes more tense, and often seems to project above an inch beyond the distended line; in which case it descends almost to the pelvis, being generally situated somewhat to the right of the mesial line, and on a level with the crest of the ilium. This tumor is the distended gall-bladder. In both these cases, the surface of the body is of a deep yellow colour; but I have suspected that it has not been so deep when the obstruction has been in the hepatic as when in the common duct: of this, however, I am by no means confident; but if it be so, the difference must arise from the change which takes place in the bile after it gets into the gall-bladder, to which, when the obstruction is higher up than the entrance of the cystic duct, it of course never gains access."

Dr. Bright relates an interesting case of *Tumefaction of the Liver from Retention of the Bile*, and then presents us with another, which is short and to the point, and we therefore quote it.

Case.—*Tumefaction of the Liver from Retention of Bile—the Gall-bladder distended with its own Secretion.*

In the Spring of this year, Dr. B. was requested by Mr. Holding to see Mrs. T——, the subject of jaundice: but the more immediate object of our consultation was a tumor which had been discovered in the abdomen, and respecting which some diversity of opinion had arisen; though Mr. Holding himself had no doubt as to its nature.

The patient was an elderly lady, between sixty and seventy years of age, who had been affected with jaundice for several weeks. The colour was a deep yellow; the stools were white, or occasionally of a pinkish-white or drab. The urine very high-coloured, yellow, and loaded with lithic deposit. On examining the abdomen by the hand, and by gentle percussion, the liver was traced, of a large volume, going back towards the loins, and descending to the umbilicus. It was smooth and tense, but not hard; and, following its margin towards the right side, and between the umbilicus and the crest of the ilium, a large rounded projection was to be plainly traced; which, in connection with the other symptoms, Dr. Bright had no hesitation in pronouncing to be the fundus of the gall-bladder. The symptoms by which the disease was chiefly marked, besides those already noticed, were anorexia, flatulency to the utmost degree, occasional vomiting, and considerable depression of spirits. Pressure made upon the liver was not immediately very painful, but left a wearing pain for some time after an examination.

No permanent advantage was obtained from remedies, and, at length, the patient sank.

*Dissection.*—On opening the abdomen, the liver was seen descending below the ribs, and the gall-bladder projecting from beneath it. The gall-bladder was not of dark colour; but was so thin, from long distention, that, while trying to raise it, it burst, and a large quantity of light dirty-yellow glairy fluid escaped. It was therefore obvious that the distention of the gall-bladder depended on something else besides pressure on the common duct; and it was presently found that a biliary calculus was impacted in the cystic duct, so that nothing could obtain an entry into the bladder, except its own secretion;—but this would not account for the jaundice: however, this was also soon accounted for, by the entire obstruction of the common duct by induration of the head of the pancreas.

Dr. Bright remarks:—" One practical point is suggested by the examination in this case. I refer to the caution inculcated by the state of attenuation to which the gall-bladder was reduced. It actually gave way under manipulation: and the same might have happened during life; in which case, peritoneal inflammation would have been almost infallible. And this struck me the more, because I had several times, during my attendance, taken the tumor in my hand, and made gentle pressure upon it as upon an elastic bottle; observing, that if I dared to make bold pressure, it felt as if I might possibly overcome the obstruction to the duct."

Two other cases of distention of the gall-bladder are related; and the observation is made that, occasionally the gall-bladder loaded with calculi is brought into a state of suppuration; and in this way, adhering to the parietes, forms an external abscess, and the calculi are discharged. In this case a tumor generally presents itself near the margin of the ribs.

4. *Hepatic Tumor from Chronic Hypertrophy of the Organ.*—"There is a state of disease," says Dr. Bright, "into which the liver is very apt to pass, when it has been long over-stimulated by habits of intemperance. The whole structure becomes uniformly changed, so that the appearance it presents is that of a yellow granular substance, like a coarse-grained sandstone; and at one period of the disease the whole organ is greatly enlarged. Whether it sometimes contracts in a later period, I am not quite sure; but if it does, it then passes into a state approaching to the hob-nailed liver: at all events, at the period of which I speak, it forms a large hepatic tumor, of a smooth character; for the granules of which it is composed are not perceptible through the parietes, which are usually, in this form of disease, rather loaded with fat, than reduced by emaciation."

Two cases are given of this affection. We proceed to—

5. *Hepatic Tumor from Fatty Degeneration of the Liver.*—"That very peculiar change to which the liver is subject when its whole substance seems converted into a mass of fat, supported in its form by the usual vessels and cellular membrane, has been known for many years, and has particularly attracted the attention of the French pathologists, who have traced it as connected in many cases with the phthisical diathesis more or less developed. I am not aware, however, that any one had pointed out a diagnostic mark of its existence during life, till Dr. Addison took up the subject, in a communication to these Reports. And to this I must refer; as I introduce the disease in this place only as affording one instance of hepatic tumor; which, however, is not a constant attendant on the disease in its early stages."

We are presented with three cases of the fatty liver. We shall select one.

CASE.—*Fatty Change in the Substance of the Liver.*

A young lady, aged 17, was in apparent excellent florid health in November 1839; except as regarded the catamenia, which came at twelve years of age, and were never regular, being frequently absent for six months at a time; but it was observed that she had grown remarkably stout. In November, she first began to feel pain in the bowels, particularly about the right iliac region. In January, she went to Brighton, on account of a disease which had taken place in the first phalanx of the great toe; and while there, diarrhœa came on to such a degree, that for twelve weeks she never had less than six or eight stools in the day; and she generally experienced a little pain in the right iliac region, and some griping over the whole abdomen.

Dr. B. first saw her on the 17th of July. She was much emaciated, yet the abdomen was not so much so as the rest of the body. Dr. B. could feel what appeared a glandular body, low down in the right iliac region, probably near the head of the colon.—Tongue red, with some elongated papillæ: stomach so irritable, that she vomited almost all her food: pulse, from 100 to 120. He saw six stools which had been passed that morning; most of them were of a remarkably healthy, brown, feculent appearance; scanty, loose, but not watery, with some small lumps in them; and in one or two, *might* be the treacle-like tinge, which a slight admixture of blood sometimes presents. Dr. B. made trial, in addition to the many remedies which had been already used without success, of small doses—first, of sulphate of copper; then of chalybeates combined with astringents: but the good effects produced were very temporary: and although at one time, on a diet of mixed food not prescribed by her medical adviser, she appeared to lose in a remarkable degree the irritability both of her bowels and her stomach, so that for two days she had neither vomiting nor diarrhœa, yet this apparent improvement passed off; and the diarrhœa returning with increased violence, she died the last day of August.

*Dissection.*—On laying open the abdomen, the omentum was seen, by no means destitute of fatty matter, spread over the abdomen, and attached at one part in the right iliac region. The liver came at once into view, of a yellow drab colour, and much enlarged: it descended at least three inches below the cartilages of the ribs, and across the whole scrobiculus cordis, quite to the spleen on the left side: it ascended to the interval between the third and fourth rib on the right side, and occupied a considerable space in the left hypochondrium. It was a perfect specimen, throughout, of the advanced fatty liver. The scalpel was covered with grease; a portion, on applying heat, yielded drops of fat, and made an oily stain on linen; and a piece of the liver, thrown into water, floated readily. A considerable quantity of blood flowed from the incisions of the liver. The gall-bladder contained about two drachms of healthy bile, and a gall-stone, of the size of a small filbert, of crystalline cholesterine.

*The Intestines.*—The whole peritoneal covering perfectly healthy, smooth, shining, and free from any effusion: but on following out the course of the intestines, they came, in the last two or three feet of the ileum, to some dark discoloured spots, where the bowel was contracted, evidently corresponding with internal ulceration; and on arriving at the termination of the ileum in the cœcum, the intestine formed a mass of the size of an egg, in which the vermiform appendage was glued with a portion of the omentum to the cœcum. On laying open the intestines, they found about ten separate ulcers in the lower part of the ileum, some of which embraced the whole calibre of the tube; but the chief ravage was about the ileo-colic valve, which was involved in a mass of ulceration, as was the pouch of the cœcum, and the cavity of the vermiform process. The other parts of the mucous membrane were healthy; and the whole lining membrane of the colon was perfect, except one small ulcer about the sigmoid flexure: and in the rectum the membrane was red, but not ulcerated. It was obvious that much tubercular deposit had taken place in the ulcerated patches, previous to their ulceration; for some such deposits lay around them, to which the ulcer had not extended.

The mesentery still contained some fat; and the glands were much enlarged, some of them going into a state of softening and suppuration. One small tubercle was detected in the substance of the right kidney.

The lungs presented some traces of tuberculous matter.

Dr. Bright observes:—"In this case, there can be no doubt that percussion would have yielded a dull sound over an unusual extent of the upper part of the abdomen, as it did in the former case. Indeed, the similarity was so striking, that we ought almost to have inferred the nature of the hepatic enlargement. Such a diagnosis, however, should always be given with caution; although, in a case of decidedly irregular catamenia, with obstinate diarrhœa, and a large smooth tumefaction of the liver, the probability would be greatly in favour of this form of disease; and more particularly before the meridian of life, for I have more than once had reason to believe that the state of amenorrhœa was connected, either as cause or effect, with the existence of fatty liver. It may be a matter of surprise that I did not detect the disease by that state of skin pointed out by Dr. Addison; but in the distressing state in which the patient was, no striking peculiarity in this respect was observed."

6. *Malignant Disease,* for the most part, induces tumors of the irregular form, yet it occasionally happens that it is otherwise, more particularly when the disease develops itself very generally through the structure of the organ, forming a great number of small and almost confluent tubera, and thus producing an even surface. Of this we have a sample.

CASE.—*Malignant Disease producing a regular smooth Enlargement of the Liver.*
April 13, 1834. Dr. Bright was requested to see Mrs. S., who had been delivered of a living and healthy child two days previously. The abdomen had scarcely diminished since parturition. On examination, a hard smooth tumor could be distinctly traced, occupying all the upper part of the abdomen; rendering the lower half of the right chest dull, and descending some way below the umbilicus. Although the situation of the tumor pretty plainly pointed it out as the liver; yet some who examined it, finding it pass quite over to the left side, had been inclined to think that the spleen was also involved in the disease. The uterus was also distinctly felt in the pelvis. The skin was sallow: there was no peritoneal tenderness. She continued to get lower, and died on the 10th of April.

*Dissection.*—About two quarts of yellow serum in the cavity of the abdomen. The lungs were pressed upward by the liver, which, in the recumbent posture, and with the lungs empty in death, had encroached on the chest, as high as the fourth rib. The liver, when the chest and abdomen were both laid open, occupied full half of both the cavities: it spread from one side to the other completely, and extended from the fourth rib to considerably below the umbilicus. It was diseased in almost every part; presenting, on its surface, circular white masses, which were not the least elevated, but rendered the whole mottled with white spots, varying from the size of a shilling to a pin's head, irregularly distributed, but occupying by far the larger proportion of the whole. The peritoneum itself was very little influenced. The gall-bladder contained a small quantity of green bile. Pancreas healthy. Spleen healthy, but large.

### HEPATIC TUMORS OF IRREGULAR FORM.

The tumors of this class are, abscesses in the liver, in various conditions; some other results of chronic inflammation; hydatids; and the different forms of malignant disease.

### HEPATIC TUMOR FROM ABSCESS.

When inflammatory affections of the liver have gone on to the formation of abscess, it depends entirely upon the situation in which the suppuration takes place, whether it produces a tumor externally or not. In general, however, some enlargement of the liver follows almost necessarily: and if the abscess does not point sufficiently, or if it be placed completely under the vault of the diaphragm, still it pushes the liver down, so that its margin is perceptible some way below the ribs: this produces an even smooth enlargement, rather than an irregular tumor; and usually the dulness of the right side of the chest extends higher than in health. When the abscess is so situated as to point externally, a distinct tumor is induced; sometimes protruding the ribs, and even pointing between the costal spaces; at other times appearing either immediately below the cartilages, or at some distance from them; the situation, of course, varying according as the right or left lobe is affected. A tumor arising from such a cause is easily to be traced as connected with the liver, of which it obviously forms a part; the dulness, on percussion, being continuous, as well as the resistance on pressure. The resistance, however, is not very great, as the whole organ rather gives way under pressure; and the sensation to the touch is comparatively soft, or it yields an elastic tenseness. More or less pain, and that often acute, is experienced when pressure is made; and generally symptoms of an active, febrile, and inflammatory character have preceded the appearance of such a tumor. It must however be borne in mind, that the approach of an abscess in the liver is often so obscure, and so insidious, that the inflammatory symptoms have sometimes not been recognised; or have, if not overlooked, frequently been ascribed to other organs; so that the appearance of the tumor has first suggested the mischief which had been going on. Its progress, too, has often been insidious; and an abscess has become chronic, producing an enlargement of a still more striking kind than I have just spoken of, remaining for months as a tangible tumor, almost defying diagnosis; and at length destroying life, by wearing out the constitutional powers, or by some accidental effusion of the pus into the peritoneal cavity. Still further than this, however,—an abscess of the liver may produce an uneven lobulated condition of the liver, possibly by absorption of the pus; or, more probably, by the escape of the greater part of it through the gall-ducts, and a consolidating change of what remains, which becomes insulated in the thickened cellular membrane. What we then find, is, a deep cicatrix marked on the surface of the liver: and when we cut through this, a yellow deposit, of a more or less purulent character or of a chalky consistence, is lodged at the bottom. Such cicatrices are not matter of doubtful existence, but deep and tangible indentations on the surface of the liver; and though generally concealed from the touch by the ribs, yet if the liver were brought down below the ribs by its own enlargement or by external pressure, the nodulated liver would present a very perplexing variety of tumor, which would most likely be mistaken for malignant disease, till sufficient time had elapsed to prove its comparative innoxious nature, and its little disposition to increase.

Two cases are detailed, both interesting. We regret that we have only room for one, the more curious of them.

CASE.—*Deep Cicatrices of the Liver from former Abscesses.*

Dr. B. was requested to meet Dr. Budd and Mr. Bell, in the case of a gentleman sinking under the effects of granulated kidneys with albuminous urine. He had made several voyages to India in his youth; but had retired from that service above fourteen years. During his Indian voyages and residence, he was supposed to have suffered from liver disease: and he has always asserted that he was sure his liver was still diseased: one reason for which belief had been, his great tendency to dysenteric diarrhœa, and derangement of the bowels.

Dr. B. was present at the post-mortem examination: and the first thing which drew attention, was the singular appearance of the liver; which was divided by several deep fissures, some of them a full inch in depth, rendering the whole liver irregularly tuberculated. These fissures were the cicatrices of abscesses; and on cutting through them, we found at least twenty small deposits of puriform matter, contained in little cyst-like cavities formed by the induration of the cellular membrane of the liver; and some of these deposits, though apparently locked up in these cavities for several years—for there was no sign of recent action—still retained the character of most perfect recent pus.

As Dr. Bright observes, this case is instructive, as shewing the frequent termination of hepatic abscess, and the way in which the remaining portions of pus may become so insulated as to be productive of little or no inconvenience, locally, or on the system.

IRREGULAR SURFACE OF THE LIVER INDUCED BY CHRONIC INFLAMMATION.

" Under this head I would arrange the numerous cases in which, from contraction of the cellular membrane, the liver becomes deformed and lobulated, either in large proportion, or in that more uniform manner which marks the hobnailed liver. As in this form of disease the liver is generally contracted rather than enlarged, we are frequently deprived of an opportunity of ascertaining its state with certainty, though the general symptoms frequently lead us to correct diagnosis. These conditions of the liver are very apt to be marked by the effusion of blood into the stomach and intestines, leading to most severe and repeated hæmatemesis, as was very well pointed out by Dr. Law of Dublin; and also to serous effusion into the peritoneum. It is owing to this last circumstance that we are often led to search for the liver, and to detect it even when its bulk is rather diminished than increased; for as the ascitic patient lies on his back, if the liver be indurated and contracted, it tends to gravitate of its own accord, from its attachments; and thus, falling downwards and forwards, sinks, suspended under a certain quantity of the serum: and thus we find it below the margin of the ribs, so as to be plainly felt. For this purpose the attention of the patient must be drawn away, if possible, to prevent the almost involuntary tension of the muscles; and then, the points of the fingers being placed on the surface, by a quick movement are brought down with the integuments so as to displace the serum and receive the impulse of the liver; and then, taking advantage of a favourable moment, the irregularities of the surface may be felt. Thus I have before me cases where the abdomen is described as loaded with serum, and the liver to be distinctly felt below the ribs; and yet, when the examination was made, after death occurring in a few days, the liver is stated to be rather small, its whole surface granulated, and its texture hard and unyielding.

There are a few other cases of tumors, of a more casual kind, formed on the surface of the liver; as, cartilaginous deposits; and even bony tumors, the result of morbid actions, which are generally not progressive. The possible existence of such tumors should be carefully borne in mind, as pointing out the propriety of abstaining from the use of violent remedies for the removal of any internal tumor, whose stationary condition, and the little effect it produces on the constitution, seem to point it out as less likely to prove injurious than our efforts for its removal, which at length will probably be of no avail."

### IRREGULAR TUMOR OF THE LIVER FROM MALIGNANT DISEASE.

Dr. Bright observes that the different forms of malignant disease must be considered amongst the most common sources of hepatic tumor. We frequently find the liver alone the seat of such disease; but, on the other hand, we still more frequently have instances of the successive or simultaneous attack of several organs. When the disease is confined to the liver, the situation of the tumor is most easily ascertained; for as there is no complexity of diseased organs, we are of course less liable to be led into error: but the nature of the disease is often more easily ascertained when other parts are affected; more particularly such as present facilities for external examination, as the mammæ, the uterus, or the superficial glands; which are all very frequently implicated, and, being far removed from the liver, afford no room for confusion: and the very circumstance of the organ being involved, strengthens the probability of the malignant nature of the disease. On the contrary, however, the greatest sources of difficulty, as to the situation of the disease, occur when the right kidney, the ascending colon or its arch, the stomach, the pancreas, or the peritoneum, are involved in the same disease with the liver.

Malignant disease varies considerably in the forms it assumes; but in general, when developed in the liver, shews itself as rounded masses or tubera, approaching more or less to the spherical shape. Dr. B. regards the malignant growths as generally originating in the cellular membrane connecting the essential portions of the organs in which they are found; often, at first, merely displacing the structures which are employed in the proper function of the organ, and interfering therefore but little with its duties; but ultimately entering so minutely into these structures, as to effect an apparent conversion, or an obliteration of the whole. Three very distinct varieties present themselves;—cerebriform disease, and that running into fungus hæmatoides; the hard scirrhus; and the melanosis. Or perhaps the hæmatoid form of the disease might deserve a separate place; making, in that case, four varieties.

Of these, the cerebriform, with or without the hæmatoid, is often the most rapid in its growth, and forms the largest and most distinct tumors in the liver: next to that, the melanosis: and the scirrhus, though apt to attack a great many points simultaneously, is the slowest in its progress, and often the least easy to recognise as a distinct irregular tumor.

Dr. Bright relates three cases of Cerebriform Growths in the Liver, which we must pass by, contenting ourselves with only the following note of them:—*that* all three of the patients had been invalids, and suffering from abdominal pains for that length of time, and some with severe occasional aggravations; and in each, the attack which preceded death, and in which the hepatic tumor was first detected, was one of intense pain in the right hypochondriac region. They all died worn out by exhaustion and suffering.

The disease had, in each, shewn itself by tubera of considerable magnitude; and in two, had shewn a tendency to attack the glandular structures about the head of the pancreas and the pylorus: and had not in any case displayed itself by affections of the serous membranes, which we find very common in the most strictly scirrhous form of disease.

The next case is one of Tumor in the Abdomen, from Scirrhous Tubera in the Liver—Peritoneum and other Organs affected.

The next—Liver converted into a Scirrhous Mass, so contracted as to form no External Tumor.—Uterus scirrhous.

We introduce the next.

Case.—*Scirrhous Tubera of the Liver.—Mamma and Ovaria diseased.*

Mary Read, aged 45, was admitted, under the care of Mr. Morgan, with scirrhus of the right mamma. She was unmarried, of sallow complexion, and spare habit. Ever since she can remember, even when young, she had a small hard tumor under the right nipple, occasionally accompanied by pain. Menstruation always regular, to the present time. Within the last three years the tumor has increased; with more pain in the part, and derangement of the general health. The breast very hard, moveable, and, in some parts, seems to contain fungoid cysts. There is manifest irregular hardness in the region of the liver; and therefore no operation was admissible.

She shortly became the subject both of ascites and anasarca, and died March 6th.

*Sectio Cadaveris.*—The liver was seen of great size, and universally pervaded by carcinomatous tubera, from the size of a grain of rice to that of a plover's egg. They all assumed a spherical shape, forming circular spots upon the surface, which, in most parts, touched each other, and in some, pressed each other out of shape, occupying a very large portion of the surface. The circular patches were of a whitish flesh colour, depressed towards the centre, and scarcely elevated at their circumferences, and marked with radiated vascularity, protruding from their centre. They were harder and more elastic than the surrounding liver; but could not be completely separated from it, as their edges, though defined to the eye, seemed to be not only strongly attached, but actually to amalgamate with the liver, as if the morbid deposit were insinuating itself between the acini. The liver itself was rather soft, of a light colour, and some parts stained with bile. The coats of the gall-bladder were about a quarter of an inch thick, somewhat resembling a scirrhous stomach, and very much contracted.

The left kidney had a fungoid growth upon its surface. In the left ovary was a well-marked apoplexy of a Graafian vesicle: in the right ovary, a fungoid growth. The lumbar glands partook of the malignant disease.

The two succeeding cases are samples of Melanosis—the first, Large Irregular Tumor, from Melanosis of the Liver—the second, Melanosis occupying the Liver very extensively; very slight Jaundice before death.

We shall introduce the first.

Case.—" December 14, 1839.—I was requested to see a lady affected with a great enlargement of the abdomen. She was a married woman, and had lain-in fourteen months before, at which time the abdomen was not discovered to be diseased; nor was it till eight months ago that the tumor was detected. Within the last week effusion had taken place into the peritoneum. I found her greatly emaciated; her complexion scarcely sallow, certainly not jaundiced. She was as large as a woman near the full time of her pregnancy. On examination, I found a hard nodulated mass, extending quite to the pelvis, and pretty obviously continuous with the liver. The ribs on the left side rather raised, and percussion dull. Examining this extensive tumor as carefully as I was able, I could not satisfactorily account for one or two hardened masses situated in the left iliac region with the liver; and I therefore concluded that the disease had been communicated to the peritoneum or omentum. I entered in my note, that ' I perceive one black spot, which I consider a small melanotic tuber, under the skin on the abdomen; and I suspect that the disease will be found of that character.'

The swelling increased. The œdema of the legs was so great, that the cuticle gave way. She greatly emaciated; and sunk, exhausted, on the 11th of January: yet within forty-eight hours of her death she had been able to come down stairs and join her family.

*Sectio Cadaveris.*—On removing the parietes, and opening the chest, the liver was seen, as a black mass, extending from the fifth rib to the pelvis and into both lumbar regions. This was everywhere pervaded by melanosis; in some parts assuming rounded forms, but generally appearing to percolate between the acini, without attaining any fixed form, or being moulded by the cellular tissue in which it was deposited. Mingled with this black matter were many small white tubera of a scirrhous character; and in one part of the convex surface a space of several inches had the appearance of a porphyritic granite, from the intermixture of the white and black. The gall-bladder contained but little bile: a few small melanotic glands on the mesocolon: the spleen and kidneys healthy. A few very small glands pervaded by melanosis were seen buried so deep in the integuments as not to shew themselves on the surface. One melanotic gland on the pericardium: one small one on the heart itself. There was decided melanotic deposit in the cancellated structure of the sternum, about its juncture with the first and second ribs.

The upper part of the lungs was spotted with melanosis, in round spots, not resembling the ordinary pulmonary blackness."

The last case is an instance of—Extensive Malignant Disease, very rapidly implicating both the organs of the Chest and the Abdomen.

Dr. Bright concludes by observing,—

" The cases and observations which I have thus thrown together, may be considered as forming an outline of one very important class of abdominal tumors; and though many of them would seem to point out the difficulties of diagnosis, yet I trust, as a whole, they may rather serve as an assistance and an encouragement to our endeavours, in this essential pursuit."

### WILLS HOSPITAL, PHILADELPHIA.

REPORT OF CASES TREATED DURING THE MONTHS OF OCTOBER, NOVEMBER, AND DECEMBER, 1839. By ISAAC HAYS, M.D.*

1. *Inability from Partial Amaurosis to distinguish certain Colours.*

Dr. Hays relates the particulars of an interesting case, and makes it the vehicle of some equally interesting observations. We shall merely mention the leading feature of the case.

An unmarried girl, aged 20, had suffered two attacks of cerebral disease, one in the spring of 1837 the other in the winter of 1837-38. After recovery from the first attack, objects for a time appeared to her double. The second attack left her entirely blind, in which condition she continued for four months. After this her sight began to return, and at the period of her admission into the hospital, in February, 1839, she was able to read large print. In May, she came under Dr. Hays's notice.

Whilst examining her at this time to ascertain the degree of vision she possessed, her reply to one of our questions led us to suspect that she was unable to distinguish colours. When asked whether she could see the figure in her dress, which was a calico one with red spots, she replied " Yes, I see the *brown* spots." Our attention thus directed to the subject, we soon ascertained that while she could distinguish forms, even of small size, with accuracy, her perception of colours was exceedingly imperfect. Repeated and careful investigations during this and on several subsequent occasions, satisfied us that the only colours which she knew with certainty were *yellow* and *blue.* Nearly all other colours she termed brown, or hesitated to name, designating, however, their shades or intensity of colour accurately. Thus a deep red she called a dark brown, a bright green a light brown, and a very pale pink a very light shade of brown.

We exhibited to her both by day and by candle light, a number of colours and have them now in our possession with the names she bestowed on them. With the exception of yellow and blue all the other colours were named with much hesitation, and some only after our insisting on her doing so, and she then manifestly named them by guess.

The treatment adopted was that calculated to remove cerebral fulness; and, under this, her sight improved, and with it the ability to distinguish colours. By the end of October, we are told that she distinguishes all the primitive colours readily and names most of the secondary ones as correctly as could be expected from one of her moderate intelligence, with the exception of violet: this last she seems always at a loss to name.

The remarks of Dr. Hays embody most of what is known upon the subject of the inability to distinguish colours. We think our readers will be well pleased if we quote them.

The feature, says Dr. Hays, of most interest in this case is the inability to distinguish colours. This is, we believe, the first example hitherto recorded of this inability having resulted from disease, or been co-existent only with it. As a natural defect, the power of distinguishing forms being perfect, it is not rare. Several instances of this have come under our own observation, and not a few others have been mentioned by writers. Such of these last as have been recorded with sufficient details to furnish data for comparison, viewed in connexion with the case we have recorded, lead to conclusions which it may not be uninteresting to notice.

* American Journal, Medical Sciences, August, 1840.

1. As a natural defect, inability to distinguish colours may exist in different degrees.

2. In the worst degree, the individual is able merely to distinguish shades,—the perception of colour is entirely absent. Examples of this are afforded in the two Harris's, who could distinguish a striped riband from a plain one, but could not perceive the difference between any one colour and another, except as darker or lighter, and in Dr. Elliottson's second case.

3. In the next degree, the individual can distinguish only a single colour, and that colour is always yellow. Thus Dr. Butter states that Robert Tucker knew to a certainty *yellow* only; and it appears that the boy whose case is recorded by Dr. Nicholl was in the same condition. Now it may be called to mind that Mary Bishop states when her sight improved the first colour she recognised was yellow.

It may be mentioned here, as connected with this subject, that we noticed a similar phenomenon in the case of a lady whom we attended for amaurosis in the winter of 1837-8. This patient, who was quite blind, began to recover her sight, and among the early evidences of improvement she mentioned, was her ability to distinguish shades of colour, as the stripes in a Venetian carpet; she could not perceive, however, a single colour. When further improvement took place she stated that she could recognise the *yellow* colour of a large looking-glass frame. A relapse then took place, from which she has not since recovered.

4. We may consider as the next degree of this defect where the individual can recognise two colours only; and these seem to be always *yellow* and *blue*. This is the most common grade of this defect. Examples of it are afforded in Scott, Dalton and his brother, in the case recorded by Dr. Nicholl in the Med. Chirurg. Trans. ix. 359; in that of J. B. related in the Transactions of the Philosophical Society of Edinburgh, vol. x. p. 253; James Milne, Mr. C., Mr. Troughton, and Dr. Elliotson's first case, and Sir David Brewster's case. Mr. Scott, J. B. and Mr. C. were imperfect in their recognition of blue; in the other cases the perception of yellow and blue seemed complete.

It is remarkable that, whilst all the individuals who belong to this class of cases are able to discern yellow and blue, they cannot distinguish these colours when presented in a state of mixture. Green they do not know—they seem blind to it. They cannot perceive any difference in colour between a stick of red sealing wax and a green table-cover; between the colour of the scarlet fruit of the Siberian-crab and the green of its leaves, &c., &c.

So it was with Mary Bishop: whilst able to detect yellow and blue she could not see the difference in colour between the red roses and their green leaves. It was not until her eye had become sensible to red that she could distinguish green.

5. It seems probable that individuals who are able to recognize *accurately* the three primitive colours, can also distinguish the secondary ones. To future observations must, however, be left the decision of this question. But persons whose perception of red is imperfect do not accurately discriminate the secondary colours.

As the imperfection in vision we have been noticing is a very curious one, it may be allowable here to call attention to some further facts connected with it.

It must be remarked that whilst those who labour under this defect naturally are unable to distinguish certain colours, though of the most vivid kind, they can discriminate any marked difference in *shades* or degrees of colour, and can see minute objects often with perfect distinctness. It occurs in persons whose point of vision is natural, as was the fact in most of the cases on record, and also in those who are far-sighted, as Mr. Nicholl's fourth case and Mr. Colqhoun's second case; and in those who are near sighted, as in Mr. Dalton.

This defect appears often to be hereditary, or at least to prevail in certain families. Thus Harris had two brothers who were unable to distinguish colours, while two other brothers and sisters, as well as his parents, had not this defect. Scott's father and one sister had the defect; his mother and another sister were free from it; but his mother's brother had it. The former sister had two sons, both labouring under the defect. Scott had two children who were able to distinguish colours. In Nicholl's first case the mother and father and his four sisters were free from this defect, but his mother's father had it. This last had two brothers and one sister; one brother had the defect, the others not. In Dr. Nicholl's second case several of the family were similarly affected. Mr. Dalton had a brother who laboured under the defect, and he mentions that he knows of a family of six sons and one daughter, in which four of the sons were unable to distinguish colours. Tucker's maternal grandfather had this defect; Wardrop states that several branches of a noble family in Great Britain have been remarkable for having it; and we know of a family in this country similarly circumstanced.

We have often noticed that persons affected with cataract, who were unable to discern the *form* of objects, in consequence of the irregular refraction of some of the rays of light and the interception of others, could distinguish generally, very accurately, *colours*. Connecting this fact with the inability to perceive colours while forms could be discerned, as observed in Mary Bishop and some other cases of amaurosis, it occurred to us that we might derive from this a means of diagnosis between the two diseases. Subsequent investigations have not confirmed this idea. The subject may, however, be worthy of a more extensive examination than we have bestowed on it.

We fancy, it is hardly worth while to notice the theories that have been proposed to account for the phenomenon. The most probable is that which places the defect in the sensorium.

---

### NEW YORK HOSPITAL.

### Select Surgical Cases. Reported by George Buck, M.D. Surgeon of the New York Hospital.*

We are glad to perceive that our contemporary of New York has devoted a distinct section to Hospital Reports. This is as it should be. If the medical officers of large hospitals, whether in this country or abroad, would publish at stated and regular periods the compressed results of their experience, what a mass of clinical wealth would be diffused throughout the medical world. We would take, however, the liberty of hinting that long cases spun out with diurnal details, and containing only facts that every body knows, are not the sort of hospital report that is wanted. Such disgust rather than attract, and are read neither by old nor young. Select cases and general results are, as it seems to us, what are required. But to the paper before us.

### Injuries of the Head.

1. *Condition of a Trephine-hole in the cranium, two years after the operation.* A boatman, aged 22, was admitted Aug. 22, 1837, with compound fracture of the cranium with depression. On the fifth day after the injury, convulsions, chiefly of the left side, occurred, the trephine was applied, many pieces of bone

---

* New York Journ. of Medicine and Surgery, Oct. 1840.

removed, and the patient relieved. He recovered from the accident, and died two years afterwards of phthisis. The hole made by the trephine was now examined.

The shape of the opening was an irregular triangle with rounded angles, two inches and a half in its largest diameter, and two in its shortest. The external table of the bone was rounded off at the margin of the opening, and the internal, which extended beyond it, remained sharp at its edge, from which a strong tense membrane stretched across the opening and closed it up. This membrane was evidently composed of the pericranium and dura mater, which could be raised from their respective surfaces, and traced as far as the margin of the opening, where they closely adhered to each other; its external surface was rough and shreddy from adhesions to the scalp; the internal smooth and shining; near the centre of the latter surface there was a circular depression of the size of a five cent. piece, with rounded edges, that appeared as though there had been a loss of substance in the dura mater. At this point the convolutions adhered by means of the arachnoid membrane, which was thickened. There were no bony deposits between the layers of the closing membrane.

The following case is not devoid of interest.

CASE.—*Convulsions following an old Injury of the Head; cured by application of the Trephine.*—August 22, 1839. In company with Dr. A. Welch, of Wethersfield and Dr. Fuller, of Hartford, Dr. Buck visited Mrs. M'D. aged 35 years, a person in humble circumstances, who was then recovering from a severe attack of convulsions, for which it had been necessary to employ the most active depleting means. She was pale and feeble, but able to sit up, and had been confined for several weeks. Upon careful enquiry, the following particulars of her case were ascertained. Three years previous, while living in the city of New York, she received a blow in the middle of the forehead from a stone, which stunned her for a short time. On recovering her senses, vomiting came on and continued for several hours. Two or three days after the injury, Dr. Mott saw her and removed two small portions of bone. The wound continued to suppurate for several months afterwards and then healed up. About eighteen months after the accident, she was attacked with convulsions, when far advanced in pregnancy with her third child. Bloodletting with other depleting remedies relieved her, so that she recovered her usual health and went her full time. After a respite of several months she was attacked a second time, and relieved by the same means. She was now recovering from the third attack, which had been more violent and protracted than either of the preceding; during the interval that had elapsed between it and the second attack, she has several times suffered from threatening symptoms, such as flushed face, drowsiness, and torpor, of which she had been relieved by blood-letting. She never suffered from headache, nor referred any uneasy sensations to the seat of the wound. During the attacks, her face and all her limbs were alike convulsed. She was evidently agitated by our visit, and her recollection confused; with the assistance of her husband, however, she was able to make out the order of different occurrences. The cicatrix upon her forehead presented the following appearances; it was an inch and a half in length, in the form of a narrow furrow, with its upper half lying in the median line, and its lower a little to the left of it, and terminating at the eyebrow; it was one-third of an inch in width, one-fourth in depth at its upper half, growing more shallow towards its lower extremity; there was evidently a loss of a portion of the external table of the skull, and the cicatrix of the skin was evidently adherent to the bottom of the furrow. The parts were free from tenderness or pain. The patient as well as her husband, were exceedingly anxious for relief, and willing any operation should be performed that afforded a prospect of benefit.

Our opinion concurring in favour of trephining, Dr. Fuller proceeded to perform the operation. A semi-lunar incision was made across the forehead, with its convexity directed downwards, intersecting the cicatrix near its inferior extremity, and extending an inch on either side of it. From the middle of this, a second incision, two inches in length, ascended along the median line: the angles were dissected up, and the periosteum detached from the bone. The first application of the trephine included about half an inch of the upper extremity of the furrow, and the disc removed appeared to be of unusual thickness, owing to in creased deposit of osseous matter upon its inner surface. A second disc was removed, joining the first at its lower edge, and including the remainder of the furrow; the thickness of this disc exceeded that of the first, and was nearly half an inch. It was perforated by a foramen that transmitted a vein from the scalp to the sinus; the surface of the dura mater exposed was of a normal appearance. A moderate oozing of blood took place during the operation; but no ligatures were necessary. A single suture secured the angles of the wound in their proper situations, and the edges were kept in contact by adhesive straps. Compresses of lint secured by long adhesive straps, completed the dressing. The patient after the operation expressed satisfaction at the relief it had afforded; her head, she said, felt altogether differently from what it had before. The wound healed kindly by the first intention, and in about a fortnight she resumed her accustomed occupations.

August 31st, 1840. This patient has continued to enjoy good health, without any return of convulsions; though once or twice she has had some threatening indications, which Dr. Welch has relieved by seasonable treatment.

### Injuries of the Spine.

Case 1.—*Supposed Fracture of the Fourth Cervical Vertebra; Recovery.*

Peter Gilman, labourer, born in Ireland, aged 26 years, of robust constitution and temperate habits, was admitted into the New-York Hospital with injury at the nape of the neck. On the 4th of December, 1839, he was suddenly thrown from his cart, while riding over rough ground, and struck with violence upon the back of his neck, that caused an immediate loss of the use of his arms. He raised himself, however, to a sitting posture, and with the assistance of another person, stood up and walked a short distance, when complaining of weakness, he was put upon a cart and conveyed home. On the way, he entirely lost the use of his right leg, and in part, that of his left. The second day following, this also became powerless like the other. Since the accident, his urine has been drawn off at stated intervals, and his fæces been voided involuntarily. He has been bled, blistered, and purged. On admission, the sixth day after the accident, his condition was as follows: No irregularity of the spinous processes of the cervical vertebræ could be felt, nor was any external sign of injury visible on the nape of the neck. Pressure, however, upon the spinous process of the fourth vertebra caused severe pains, that, he said, extended to the fingers and toes. Bending the head forward has the same effect, while rotating it gave him no pain. Paralysis of the lower limbs was complete. He was still able to raise his arm to a certain extent, with a sort of dangling motion, but could not extend the forearm, or flex the fingers; sensation was not impaired. Respiration was calm and easy, and performed without motion of the ribs or concurrence of the abdominal muscles. He was exceedingly restless, requiring his position to be changed every few minutes; his pulse was 64 and good. The urine drawn off at stated intervals was somewhat turbid, but without albuminous deposit; his fæcal evacuations were involuntary. Cups to be applied to the nape of the neck.

December 31st. Some improvement had taken place in his symptoms, he had become tranquil, and his general condition quite favourable. It was no longer necessary to use a catheter; by the aid of pressure with the hand above the pubes, the bladder could be evacuated. There were at times spasmodic twitchings in his lower limbs by which they were drawn up, but he had no control over them by any act of volition.

After this, a gradual improvement in all his symptoms continued, so that in the month of May following, he was able to walk about with the aid of crutches, and to pass his fæces and urine in the natural manner. A seton was kept open on the nape of the neck, and frictions with stimulating liniment employed daily, over the body and limbs. At the time of his discharge, in the following month, he was able to walk with a cane only, and was continuing to gain strength gradually.

CASE 2.—*Fracture of the Second Lumbar Vertebra, without Paralysis.*

Daniel Wilson, born in Wales, aged about 37 years, of very intemperate habits, was admitted into the hospital in a state of delirium tremens, which continued several days, until 24 hours before death. Nothing could be ascertained respecting the nature of the injury he had received, nor was its extent suspected until he recovered his reason sufficiently to make it known himself, when it was ascertained that, several days before his admission, he had jumped from a garret window 40 feet in height, in a fit of delirium. His right foot and ankle bore marks of contusion, and the extremity of the fibula was broken off. Before he regained his reason, it was impossible to keep him in bed; he was constantly going about the premises. After this, however, he began to complain of great pain, and soreness in moving his body, and as he lay in bed, his head was thrown far back. On examining his back, the spinous process of the second lumbar vertebra presented an angular projection; pressure upon it gave no pain, nor did paralysis exist.

*On dissection,* blood was found extravasated between the folds of the mesentery and into the muscular and cellular tissue, covering the lumbar vertebræ. The body of the second vertebra was broken into fragments, the first and third were uninjured; the membranous sheath, as well as the enclosed cord, were sound.

CASE 3.—*Fracture of the Skull, and of the Second Lumbar Vertebra, without Paralysis.*

Q. D., was admitted December 20th, 1830, into Ward No. 4, of the Marine Department. He had been knocked down in the evening by a carriage running against him. Several contusions of the scalp existed, besides a deep wound of an inch in length, in the axilla, from which there was but little hæmorrhage. The following morning, having recovered from intoxication, he complained chiefly of his back, and referred his pain to the lumbar vertebræ. There was no appearance of external injury or displacement; pressure did not much increase the pain. He was able to move in any direction, and even walked about the ward till the day he died; his face was flushed, and at times he was delirious. He was exceedingly averse to allow any thing to be done for him. At 11 o'clock, the third night after the injury, he was seized with a fit, and five hours after died.

*Dissection.*—There was a crack in the skull running from the vertex on the left side to the foramen magnum. The whole surface of the right hemisphere of the cerebrum was covered with a layer of coagulated blood, thickest over the inferior surface of the middle lobe, where the convolutions were softened into a pultaceous mass. The veins of the pia mater upon both hemispheres were loaded with blood. The second lumbar vertebra was fractured across the body. The anterior vertebral ligaments remained entire, excepting on the left side, where a small spicula of bone had obtruded. The cellular and muscular tissues around were infiltrated with extravasated blood.

### Immobility of the Lower Jaw. Division of the Right Masseter Muscle.

*Case.*—John Bishop, aged 19, August 19th, 1839. About 18 months before, he had had an attack of fever at the south, during which he was profusely salivated, and sloughing of the right cheek had occurred, with loss of considerable portions of the soft parts from the inside of the mouth. On recovery, he was unable to open his mouth, a band having formed, by which the jaws were kept firmly applied to each other. Unsuccessful attempts had been made to relieve this condition by dividing the constricting band, but without using means, at the same time, to force the jaws apart. Two portions of bone had been discharged.

The right cheek was full and swollen, the skin and subcutaneous cellular tissue supple and movable upon the masseter muscle, which could be felt contracting under the hand, whenever he put it in action. In a state of rest, this muscle felt hard and tense. On the inside of the cheek, a firm callous band extended from above the interval between the first and second upper molar teeth on the outside, to below the first molar tooth of the lower jaw, with a sharp unyielding edge, that would not permit the end of the finger to be insinuated between it and the outer surface of the teeth. The jaw was susceptible of a sliding motion, showing that the right temporo-maxillary articulation was movable. The upper dental arch stood a little in advance of the lower, barely allowing the blade of a table knife to be introduced between them. His voice was very little affected. He was able to take solid food by cutting it very fine, and insinuating it between the upper and lower teeth. His general health was pretty good, and he had observed no change in the condition of his jaw for a year past.

*Operation.*—A bandage of two fingers' breadth, and fifteen inches in length, was insinuated between the upper and lower teeth, and the ends tied so as to form a loop below the chin. One assistant held the head firmly, while another acted on the lower jaw, by means of the bandage, bearing down so as to put the band to be divided on the stretch as much as possible. The fore finger of the left hand was then introduced under the cheek, and the nail insinuated between the edge of the callous bridle and the molar teeth, to serve as a guide for the knife. A narrow bladed scalpel was employed, care being taken to avoid cntting near the middle of the masseter muscle, in order not to involve the parotid duct. Successive incisions from within outwards, and advancing from before backwards, guided by the sense of touch alone, were made on a level with the lower molar teeth, until the finger arrived at the last tooth; resisting bands were felt still farther back and were divided. By repeated attempts with instruments that acted as levers in prying the teeth apart, sufficient space was obtained to introduce the speculum oris, which acted with great effect in rupturing the fibres which still bound the jaws together. Proceeding cautiously, in this manner, alternately prying and dividing the resisting bands, the jaws were separated so as to allow two fingers to be placed edgewise between the incisor teeth. The whole width of the masseter muscle was involved in the incision, and in some parts its whole thickness; the knife grating as if cutting through cartilage; the hæmorrhage was moderate, and ceased spontaneously. After the operation, the patient could himself open his mouth to the extent to which the teeth had been separated. A denuded bony surface was felt on the outside, a little behind the last molar tooth, and could be traced upwards to a rough pointed extremity, which was somewhat movable. A wooden wedge was introduced between the molar teeth of the opposite side, and required to be kept in as much of the time as it was possible for him to bear it. Considerable swelling and inflammation succeeded, the following day, and continued for some time. The use of the wedge was persevered in.

After this the speculum was perseveringly employed, in order to effect dilatation of the mouth, and several bands were divided. The result was that, in March, 1840, his condition was as follows:—The right cheek is much less swollen than it was before the operation, and is soft and supple; the masseter feels hard. The forefinger can be introduced edgewise between the incisor teeth, and within these limits he has free use of the jaw, and perceives no tendency to farther contraction. The callous band on the inside of the cheek, exists very much in the same condition as before the operation, excepting that it does not advance as far forward.

The operation, then, succeeded only partially.

### EXOSTOSIS ON THE UPPER SURFACE OF THE LAST PHALANX OF THE GREAT TOE, CURED BY EXCISION.

*Case.*—William Jewell, a seaman, born in Norway, aged 20 years, of robust and healthy constitution, first noticed this disease, for which he sought relief, about fourteen months prior to the 25th of September, 1839, the date of his admission to the hospital. At that time, after walking a great deal in a pair of tight new boots, he observed a small hard lump growing under the edge of the nail of the left great toe; by keeping it paired down close, he suffered little inconvenience. It had been partially excised, together with a portion of the nail, but had grown out soon after. At the time of the operation, to be now described, the toe presented the following appearances: From beneath the anterior edge of the nail, which was cut very short, a small tumor of the size of a split pea protruded, of a reddish-gray colour and tough firm consistence, but not apparently bony at its apex. It gave him no pain, excepting when he neglected the precaution of wearing an easy shoe. From the situation of the apex at the anterior edge of the nail, I supposed its base did not extend so far back as to make the removal of the whole nail necessary, and therefore proceeded, with the view of saving the matrix of the nail. After having been previously softened in hot water, and scraped thin, an incision was made across the nail with a straight narrow bistoury, a line and a half anterior to its root, and carried perpendicular to the surface of the bone beneath. Instead of reaching beyond the base of the tumor, as was intended, the knife passed into it. It was, therefore, necessary to repeat the incision at a more remore point, which was done in the same manner, two lines beyond the root of the nail, so as to include the matrix. On coming down upon the bone, the edge of the bistoury was directed forward, and made to graze its surface, thus including the nail, with the tumor at its' origin. Its bony nature was obvious in cutting through its base, where the knife met with very great resistance. The surface from which it grew was shaved down, below the surrounding bone, until it had a healthy appearance. The resistance of the nail, perhaps, had determined the growth of the tumor forwards, by which Dr. B. was led to the oponion that it took its origin nearer the extremity of the phalanx. There was very little hæmorrhage, and the wound healed kindly under the application of simple dressings, and nitrate of silver occasionally, to repress the exuberant granulations. There was no tendency to a reproduction of the diseased growth at the time of the discharge, October 21st, 1839.

### DIVISION OF THE SPHINCTER ANI FOR ULCER OF THE RECTUM.

*Case.*—In a case of oblong ulcer of the rectum, on its posterior wall, with raised edges, of long standing, and productive of severe symptoms, the following operation proved successful.

The patient was placed on his left side, with his hips at the edge of the bed, and his thighs drawn up. The nates being stretched apart, the index finger of the left hand, smeared with lard, was passed into the anus, and upon it a probe-pointed bistoury, flatwise; the edge was then directed towards the ulcer, and in withdrawing it, was made to divide the integument, and subjacent sphincter, by an incision of an inch in length. The hæmorrhage was moderate, and ceased spontaneously. A greased tent was laid in the incision, and a compress upon it was secured in place by a T bandage. The bowels having been freely evacuated, before the operation, very low diet and confinement to his bed were enjoined, for the purpose of preventing, as long as possible, his going to stool.

